# METTL1‐Mediated M^7^G tRNA Modification Promotes Residual Liver Regeneration After Hepatectomy via Translational Control

**DOI:** 10.1002/advs.202507329

**Published:** 2025-12-08

**Authors:** Manling Huang, Shuirong Lin, Yutong Zhao, Jiale Chen, Linyuan Huang, Yuyao Liu, Zixin Huang, Dongxin An, Yifan Zhang, Zimin Song, Xi Yu, Yunpeng Hua, Jing Wang, Weiwei Wang, Yu Guo, Ming Kuang, Fang Wang, Shuibin Lin, Shunli Shen

**Affiliations:** ^1^ Department of Oncology Cancer Center The First Affiliated Hospital of Sun Yat‐sen University Guangzhou Guangdong 510080 P. R. China; ^2^ Center of Hepato‐Pancreato‐biliary Surgery The First Affiliated Hospital of Sun Yat‐sen University Guangzhou Guangdong 510080 P. R. China; ^3^ Department of Hepatobiliary Surgery Meizhou People's Hospital Meizhou Guangdong 514000 P. R. China; ^4^ Department of Ultrasound Medicine The First Affiliated Hospital of Sun Yat‐sen University Guangzhou Guangdong 510080 P. R. China; ^5^ Department of Urology The First Affiliated Hospital of Sun Yat‐sen University Guangzhou Guangdong 510080 P. R. China; ^6^ Institute of Precision Medicine The First Affiliated Hospital of Sun Yat‐sen University Guangzhou Guangdong 510080 P. R. China; ^7^ Center for Translational Medicine Institute of Precision Medicine The First Affiliated Hospital of Sun Yat‐sen University Guangzhou Guangdong 510080 P. R. China; ^8^ Innovative Cancer Research Center Zhongshan School of Medicine Sun Yat‐sen University Guangzhou Guangdong 510080 P. R. China

**Keywords:** hippo pathway, liver regeneration, METTL1, post‐hepatectomy liver failure (PHLF), tRNA modification

## Abstract

Partial hepatectomy (PHx) has emerged as a primary therapeutic intervention for end‐stage liver pathologies. However, post‐hepatectomy liver failure (PHLF), a critical complication arising from inadequate regenerative capacity of the remnant liver, underscores the clinical imperative to understand molecular mechanisms governing hepatic regeneration. Through an integrative multi‐omics analysis in a murine 70% PHx model coupled with clinical correlation studies, the tRNA m^7^G methyltransferase METTL1 was identified as a pivotal regulator of post‐resection hepatic recovery. METTL1 exhibited significant temporal upregulation following PHx, with its expression profile positively correlating with favorable clinical outcomes in surgical patients. Genetic ablation of METTL1 substantially attenuated hepatocyte proliferation and compromised regenerative capacity, whereas its ectopic expression potentiated liver regeneration through enhanced translational efficiency. Mechanistic investigations revealed that METTL1‐mediated m^7^G tRNA modification orchestrates regenerative processes by selectively augmenting the translation of Hippo pathway effectors YAP/TAZ. Most importantly, modulation of the METTL1‐YAP/TAZ signaling axis successfully promotes liver regeneration after PHx. This study elucidates a previously unrecognized translational control mechanism underlying liver regeneration, proposing METTL1 as a promising molecular target for preventing PHLF through therapeutic enhancement of hepatic regenerative potential.

## Graphical abstract

METTL1 and its mediated m^7^G tRNA modification are significantly up‐regulated after partial hepatectomy (PHx). Overexpression of METTL1 improves the efficiency of liver regeneration after PHx, increases the proliferation of hepatocytes, and accelerates the recovery of liver function. METTL1‐mediated m^7^G tRNA modification selectively governs the translation efficiency of the Hippo signaling pathway genes.

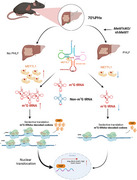

## 1 Introduction

Partial hepatectomy (PHx) is a crucial intervention for managing diverse benign and malignant liver diseases. Despite advancements in surgical techniques and perioperative management improving patient outcomes, liver parenchymal damage and insufficient future liver volume (FLV) after hepatectomy can hinder liver regeneration, leading to liver dysfunction and potentially resulting in post‐hepatectomy liver failure (PHLF).^[^ ^1^, ^2^ ^]^ PHLF is one of the most severe complications after hepatectomy, with reported incidence rates ranging from 1.2% to 32%.^[^ ^3^, ^4^ ^]^ As effective treatments are lacking, PHLF remains a leading cause of mortality after liver resection. The main feature of PHLF is the inability of hepatocytes to regenerate sufficiently and restore or maintain essential liver function. Furthermore, changes in the liver microenvironment due to acute or chronic injury can diminish hepatocyte regenerative capacity. Unfortunately, the underlying molecular mechanisms of PHLF are still poorly understood, and there are currently no effective preventive or therapeutic measures available in clinical practice.

Previous research has demonstrated that various signaling molecules play a role in regulating cell proliferation during liver regeneration. After PHx, more than 100 proteins were upregulated within the first hour, including those related to proliferation and cell cycle proteins.^[^ ^5^, ^6^ ^]^ At the same time, several signaling pathways, such as IL‐6/JAK/STAT3, HGF/MET, and TNF‐α/NF‐κB were quickly activated to promote liver regeneration.^[^ ^6^, ^7^ ^]^ Moreover, under the stress of PHx, cytokines such as TNF‐α and IL‐6 were released abundantly.^[^ ^8^ ^]^ These cytokines stimulate dormant hepatocytes to re‐enter the cell cycle and undergo mitosis at a rapid rate to generate new cells.^[^ ^9^, ^10^ ^]^ These findings suggest that the rapid synthesis of effector proteins plays a crucial role in hepatocyte regeneration after PHx. However, further investigation is required to fully understand the specific mechanisms involved.

Transfer RNAs (tRNA) play a crucial role in facilitating protein synthesis by recognizing mRNA codons and carrying the corresponding amino acids. Modifications to tRNA are essential for its stability, codon recognition, and rapid protein synthesis.^[^ ^11^, ^12^ ^]^ Recent studies have highlighted the importance of tRNA modifications in efficient protein synthesis and various biological processes. The N7‐methylguanosine (m^7^G) modification is a highly conserved tRNA modification catalyzed by the METTL1/WDR4 protein complex.^[^ ^13^, ^14^ ^]^ Studies have shown that METTL1‐mediated m^7^G modification enhances tRNA stability and expression, leading to increased translation of oncogenes and contributing to cancer progression.^[^ ^15^, ^16^, ^17^ ^]^ Notably, recent research has also revealed that METTL1‐mediated m^7^G tRNA modification plays a crucial role in promoting rapid and selective protein synthesis upon external therapeutic stress. Under radiation therapy, the level of METTL1‐mediated m^7^G tRNA modification increases rapidly, resulting in enhanced DNA ligase IV translation and increased non‐homologous end joining (NHEJ)‐mediated DNA double‐strand break repair in liver cancer cells, therefore promoting radiotherapy resistance.^[^ ^18^ ^]^ In thermal ablation stress, METTL1‐mediated m^7^G tRNA modification promotes the translation of SLUG/SNAIL and enhances the malignant potential of HCC cells, leading to resistance to thermal ablation therapy.^[^ ^19^ ^]^ These studies highlight the important role of METTL1 and its mediated m^7^G tRNA modification in the regulation of rapid protein synthesis for external therapeutic stress response. However, the physiological function of METTL1‐mediated m^7^G tRNA modification in rapid stress response and liver regeneration following PHx remains unknown.

In this study, we analyzed the translatome process in regenerating liver tissues after PHx to delineate the molecular mechanism involved in liver regeneration. Our research revealed that METTL1 expression is rapidly increased after PHx, leading to enhanced protein synthesis and cell proliferation during liver regeneration. Mechanistically, METTL1 and its mediated m^7^G tRNA modification played a vital role in regulating the translation efficiency (TE) of critical proteins in the Hippo pathway, YAP/TAZ, which in turn affected the expression of genes involved in cell proliferation and ultimately influenced the rate of liver tissue regeneration after PHx. Overall, our findings indicate that modulating METTL1 could be a promising strategy to enhance liver regeneration after PHx, potentially leading to better outcomes for patients undergoing liver surgery.

## 2 Results

### 2.1 Rapid Liver Regeneration is Associated with Better Therapeutic Outcomes After Partial Hepatectomy (PHx)

**Figure 1:**
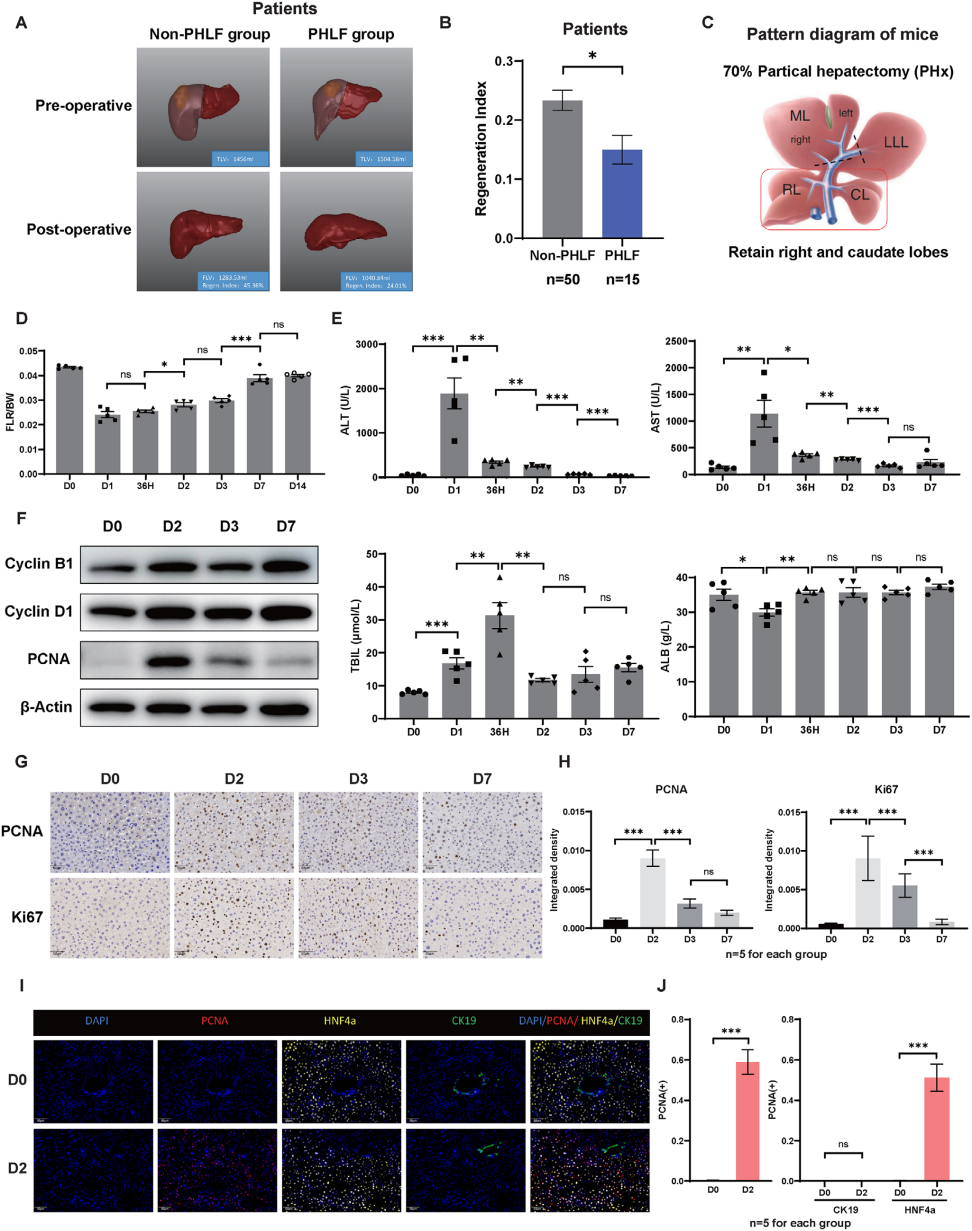
Significant Proliferation of Residual Liver after 70% PHx. A) Representative images of 3D reconstruction models of liver volume before and after surgery in patients of the PHLF and Non‐PHLF groups. B) The liver regeneration index (regenerated liver volume/TLV) was significantly higher in patients of the Non‐PHLF group (n = 50) compared to the PHLF group (n = 15). C) Schematic diagram of 70% PHx in mice: removal of the left lateral and median lobes (LLL and ML), preservation of the right and caudate lobes (RL and CL). D–F) Ratio of liver weight (LW) to body weight (BW) (D), Serum levels of ALT/AST/TBIL/ALB (E), and Western blot analysis of Cyclin B1/Cyclin D1/PCNA (F) in mice at different time points (D0‐D14) after PHx. G,H) Immunohistochemistry (IHC) staining and quantitative analysis of PCNA and Ki67 in mice at different time points after PHx. I,J) Representative images and quantitative analysis of multiplex immunohistochemistry (mIHC) staining for PCNA, CK19, and HNF4a in paraffin sections of the liver at day 0 and day 2 after PHx. β‐Actin was used as a loading control for Western blot analysis. Scale bars for IHC and mIHC: 50 µm. Significance is indicated as follows: ^*^ *P*<0.05, ^**^ *P*<0.01, ^***^ *P*<0.001, and ns indicates no significant difference.

As one of the most important organs for maintaining homeostasis, the liver has a unique regenerative ability. By studying the imaging data and postoperative follow‐up of patients undergoing partial hepatectomy, we observed that patients with rapid growth of FLV after PHx were less likely to develop PHLF than patients with restricted growth (**Figure** **1**A,B). To further reveal the specific mechanism of liver regeneration after hepatectomy, we performed a 70% partial hepatectomy procedure on wild‐type (WT) mice (Figure 1C)^[^ ^20^ ^]^ and analyzed liver tissues at different time points after hepatectomy. We observed significant residual liver hyperplasia in the early phase after hepatectomy, with the liver weight/body weight ratio rapidly increasing and returning to the pre‐hepatectomy level around the seventh day after hepatectomy (Figure S1A,B, Supporting Information; Figure 1D). Additionally, we found that the serum levels of alanine transaminase (ALT), aspartate transaminase (AST), and total bilirubin (TBIL) significantly increased, while albumin (ALB) decreased after the hepatectomy. Subsequently, the serum levels of ALT, AST, and TBIL gradually decreased, while the serum ALB level increased and recovered to an average level on the second day after surgery (Figure 1E). Furthermore, the expression of cyclin proteins and cell proliferation, as indicated by the Ki67, PCNA index scores, peaked on the second day after hepatectomy, with fewer proliferating cells observed on the seventh day (Figure 1F–H). We then utilized multiplex immunohistochemistry (mIHC) staining to further investigate the main cell types that proliferate during liver regeneration. Our data showed that the majority of proliferating cells in the remnant liver were hepatocytes (HNF4α^+^ cells), while biliary duct cells (CK19^+^ cells) minimally proliferated (Figure 1I,J). Overall, these data support that rapid liver regeneration is associated with better therapeutic outcomes after PHx, and the proliferation of hepatocytes predominantly drives liver regeneration after hepatectomy.

### 2.2 METTL1 is Upregulated after PHx and Depleting METTL1 Impairs Liver Regeneration

**Figure 2:**
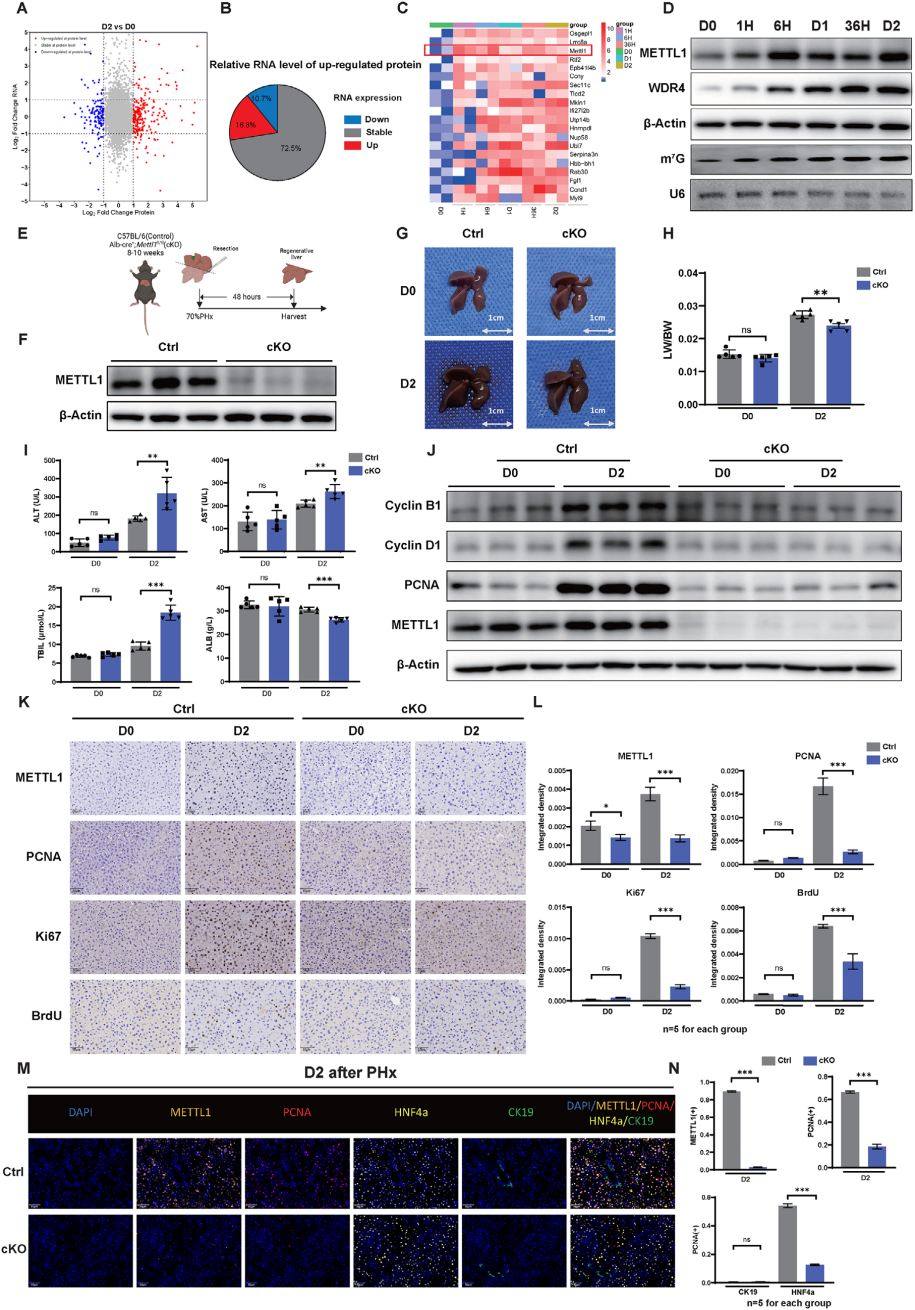
METTL1 is Upregulated after PHx and Knockdown of METTL1 Inhibits Liver Regeneration. A) Differential expression profiles of proteins and RNA between D2 and D0 after PHx in mouse liver tissues. B) Analysis of RNA levels of proteins upregulated on D2 after PHx. C) Heatmap of quantitative proteomics shows the expression level changes of proteins at different time points (D0‐D2) after PHx. D) Western and Northwestern blot analysis confirmed significant upregulation of METTL1/WDR4 and m^7^G expression from D0 to D2. E) Experimental schematic: 8–10 weeks‐old C57BL/6 (Ctrl) and Alb‐cre^+^, Mettl1^fl/fl^ (cKO) mice underwent 70% PHx, and regenerating liver tissues were collected 48 h later for further analysis. F) Western blot analysis confirmed the knockout of METTL1 protein levels in the liver of *Mettl1*cKO mice. G–J) Representative images of liver regeneration (G), ratio of LW to BW (H), serum levels of ALT/AST/TBIL/ALB (I), and protein expression level of proliferation‐related markers (J) in Ctrl and *Mettl1*cKO mice at D0 and D2 after PHx. K,L) IHC staining and quantitative analysis of METTL1, PCNA, Ki67, and BrdU in regenerating liver tissues of Ctrl and *Mettl1*cKO mice at D0 and D2 after PHx. M‐N. Representative images and statistical analysis of mIHC staining for METTL1, PCNA, HNF4a, and CK19 in paraffin sections of the liver at D2 after PHx in Ctrl and *Mettl1* cKO mice. β‐Actin was used as a loading control for Western blot analysis. U6 snoRNA was used as the loading control for Northwestern analysis. Scale bars for IHC and mIHC: 50 µm. Significance is indicated as follows: ^*^ *P*<0.05, ^**^ *P*<0.01, ^***^ *P*<0.001, and ns indicates no significant difference.

We next performed proteomics and RNA sequencing to elucidate the specific mechanism of rapid regeneration of the liver after PHx. Our data revealed a rapid upregulation of protein levels in the regenerating liver tissue two days after PHx (**Figure** **2**A), indicating that proteins were rapidly synthesized after PHx to maintain cell homeostasis and survival in response to external stimulation of hepatectomy. Notably, most of the upregulated proteins showed no change at the mRNA level (Figure 2B), implying a post‐transcriptional regulation mechanism involved. To further illustrate the underlying mechanism, we conducted proteomic analysis of regenerating liver tissues at different time points after PHx.^[^ ^21^, ^22^, ^23^ ^]^ The results demonstrated a steady upregulation of METTL1 (Figure 2C), which was confirmed by Western blot and accompanied by increased WDR4 expression and elevated tRNA m^7^G modification levels (Figure 2D). As the only catalytic enzyme of tRNA m^7^G modification, METTL1 has been widely reported to play a pivotal role in mRNA translation control and cell fate determination.^[^ ^17^, ^24^, ^25^, ^26^ ^]^ The rapidly increased expression of METTL1 after PHx suggests that METTL1‐mediated tRNA modification and protein synthesis could play a critical function in promoting liver regeneration after hepatectomy.

To further investigate the role of METTL1‐mediated m^7^G tRNA modification in liver regeneration after PHx, we constructed liver‐specific *Mettl1* conditional knockout (cKO) mice (Figure 2E,F). Interestingly, under physiological conditions, the *Mettl1* cKO mice did not exhibit differences in liver morphology or liver weight/body weight ratio compared to WT mice. However, following PHx, they exhibited severely impaired liver mass recovery (Figure 2G,H). In addition, METTL1 depletion also interfered with liver recovery, as revealed by serum levels of ALT, AST, TBIL, and ALB (Figure 2I). Consistent with this finding, a longitudinal analysis of liver enzymes revealed a more severe and protracted hepatic dysfunction in cKO mice compared to WT animals (Figure S2A, Supporting Information). Furthermore, the expression levels of cyclin proteins and proliferation‐related markers notably decreased in the livers of *Mettl1* cKO mice (Figure 2J–L). In contrast, no notable differences in apoptosis or fibrotic response were detected between the groups, suggesting that the regeneration defect is primarily due to impaired proliferative capacity rather than increased cell death or fibrosis (Figure S2B–E, Supporting Information). mIHC further confirmed that METTL1 primarily affects hepatocyte proliferation (Figure 2M,N). In line with this observation, METTL1 expression was minimal in bile duct or stromal cells during regeneration. Together, these findings indicated that METTL1 promotes liver regeneration mainly by enhancing hepatocyte proliferation, with minimal contribution from cholangiocytes or stromal cells (Figure S2F,G, Supporting Information).

Additionally, we also depleted METTL1 expression by injecting hepatotropic adeno‐associated virus (AAV8) via the tail vein. Consistent with previous reports, AAV8‐based gene delivery showed a favorable safety profile in our study^[^ ^27^, ^28^ ^]^ (Figure S3, Supporting Information). The in vivo imaging system (IVIS) demonstrated that the AAV8 was mainly concentrated in the liver (Figure S4A,B, Supporting Information). Consistently, METTL1 knockdown mice exhibited impaired liver regeneration compared with the control group (Figure S4C,D, Supporting Information), with decreased expression of cyclin proteins and proliferation‐related markers in hepatocytes (Figure S4E–G, Supporting Information). Taken together, these data demonstrated that the liver‐specific deficiency of METTL1 decreased hepatocyte proliferation and impaired residual liver regeneration after PHx.

### 2.3 Overexpression of METTL1 Promotes Liver Regeneration after PHx

**Figure 3:**
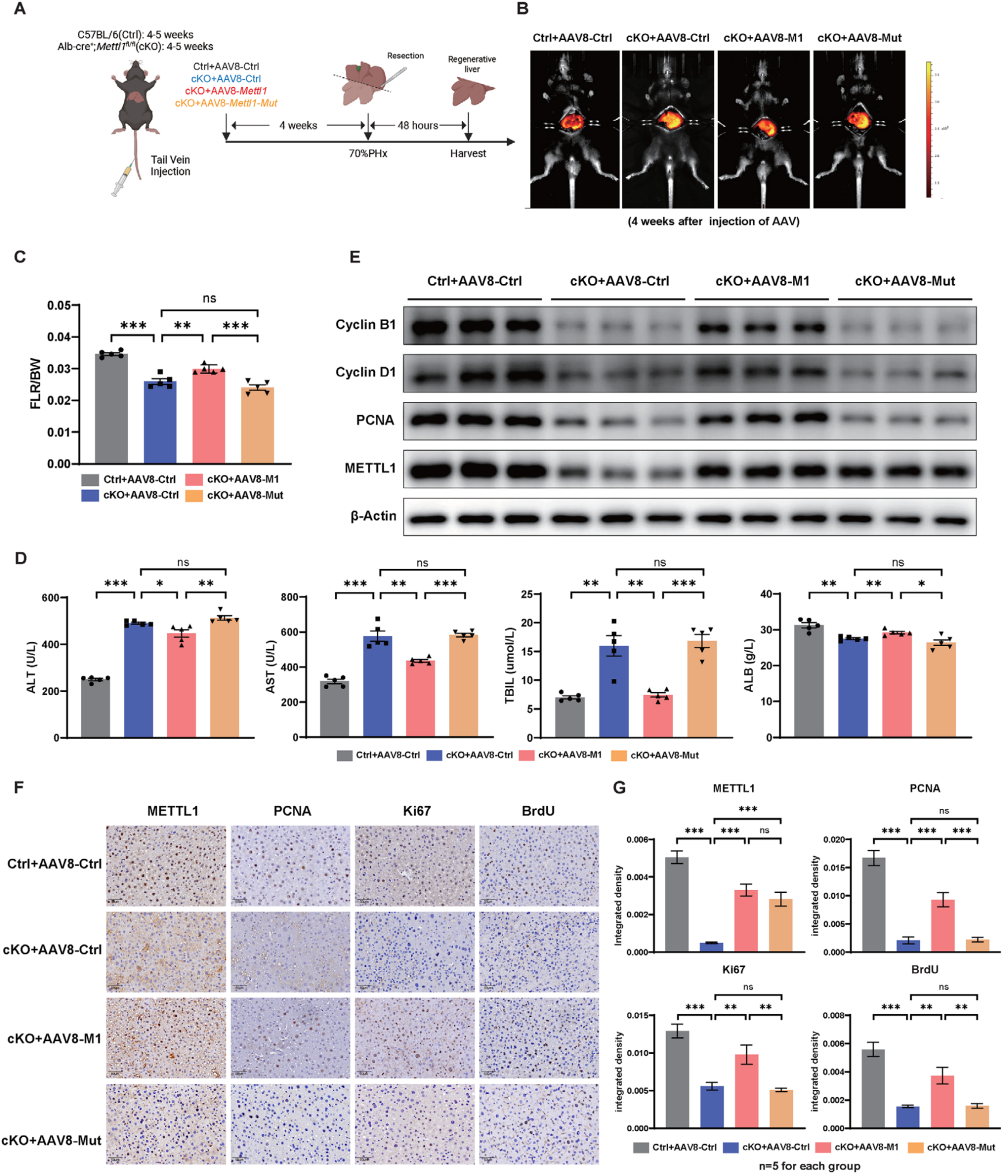
Overexpression of METTL1 Significantly Promotes Liver Regeneration. A) Experimental schematic: 4–5 weeks‐old C57BL/6 (Ctrl) and Alb‐cre^+^, Mettl1^fl/fl^ (cKO) mice were injected with AAV8 carrying *Mettl1* (AAV8‐*M1*), inactive mutant *Mettl1*(AAV8‐*Mu*t), and control ZsGreen vector (AAV8‐Ctrl) via tail vein injection, and 70% PHx was performed 4 weeks later, followed by tissue collection 48 h after PHx. B) Quantification of the accumulation of AAV8 in the liver using In Vivo Living Image software 4.5.5 (Perkinelmer, Waltham, Mass). C–E) Ratio of LW to BW (C), serum levels of ALT/AST/TBIL/ALB (D), and protein expression level of proliferation‐related markers (E) in the above four groups at D2 after PHx. F,G) IHC staining and quantitative analysis of METTL1, PCNA, Ki67, and BrdU in regenerating liver tissues in the above four groups at D2 after PHx. β‐Actin was used as a loading control for Western blot analysis. Scale bars for IHC: 50 µm. Significance is indicated as follows: ^*^ *P*<0.05, ^**^ *P*<0.01, ^***^ *P*<0.001, and ns indicates no significant difference.

We further investigated whether overexpression of METTL1 could promote liver regeneration after PHx. To address this question, we delivered AAV8 carrying *Mettl1* (AAV8‐M1) or a control ZsGreen vector (AAV8‐Ctrl) into *Mettl1* cKO mice (**Figure** **3**A,B). Notably, overexpression of *Mettl1* in cKO mice enhanced liver weight/body weight ratio after PHx compared to the control group (Figure 3C). Moreover, there was a noticeable improvement in liver function recovery (Figure 3D). Consistently, the expression levels of cyclin proteins (Figure 3E) and proliferation‐related markers, including Ki67, PCNA, and BrdU, were increased in *Mettl1* overexpressing mice (Figure 3F,G). To determine whether this pro‐regenerative effect depends on METTL1's m^7^G methyltransferase activity, we also introduced a catalytically inactive mutant (D174A/W176A) into *Mettl1* cKO mice. In contrast to wild‐type METTL1, the mutant failed to rescue the regeneration defect (Figure 3A–G). Taken together, these findings demonstrated that METTL1‐mediated tRNA m^7^G modification is essential for liver regeneration after PHx.

### 2.4 METTL1‐Mediated m^7^G tRNA Modification Upregulates Translation Activity in Hepatocytes After PHx

**Figure 4:**
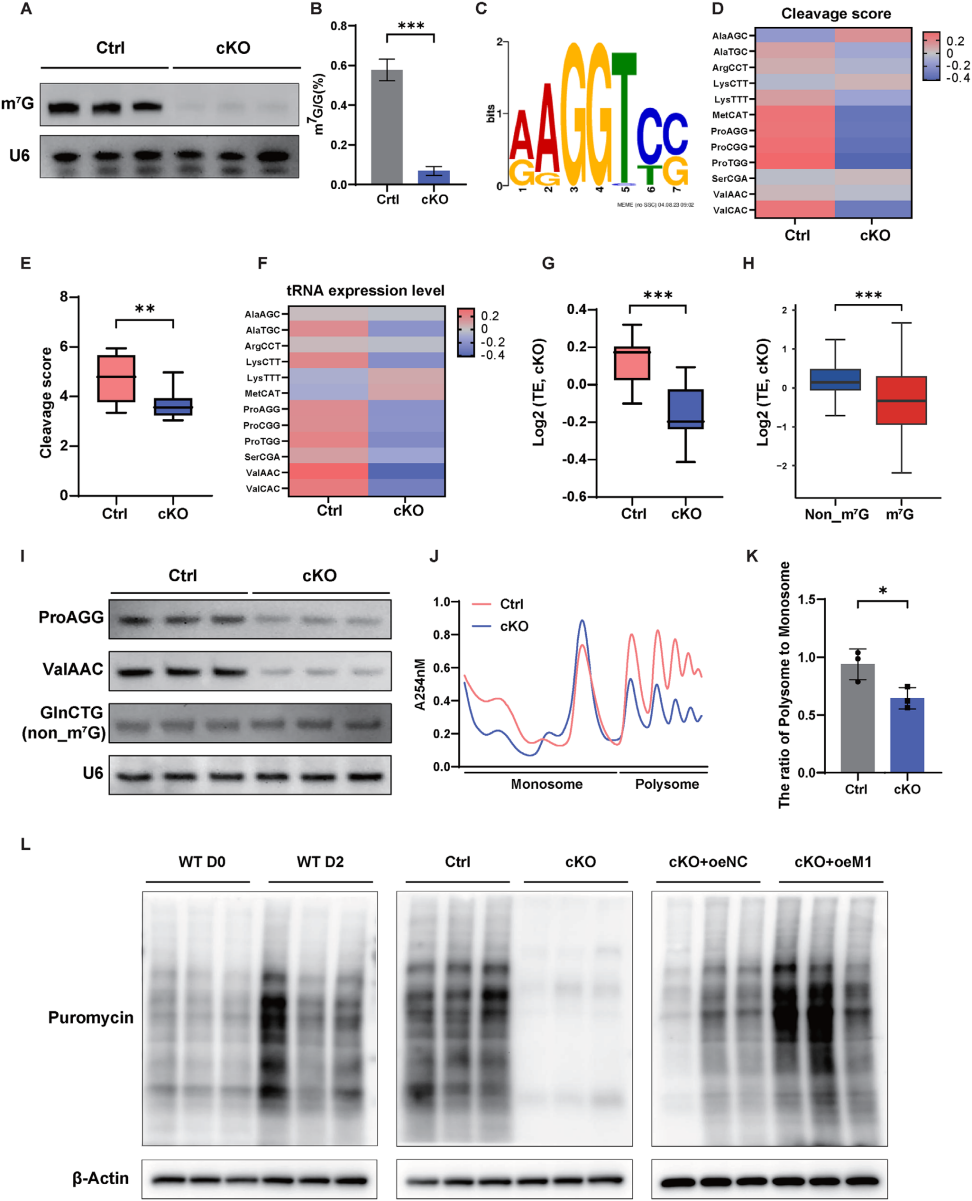
Effect of METTL1 on m^7^G Modification, tRNA Expression, and overall mRNA Translation Levels. A) Detection of m^7^G modification in tRNA of regenerating liver tissues at D2 after PHx in Ctrl and cKO mice by anti‐m^7^G Northwestern Blotting. B) Quantification of m^7^G modification level by LC‐MS/MS assay. C) The motif sequence “RGGUY” of m^7^G sites identified by TRAC‐seq. D) Methylation profiles of 12 m^7^G‐modified tRNAs identified by TRAC‐seq in Ctrl and cKO mice. E) Quantitative comparison of cleavage scores between Ctrl and cKO mice revealed a decrease in overall m^7^G tRNA methylation levels in hepatocytes of cKO mice compared to Ctrl mice. F) Expression profiles of 12 m^7^G‐modified tRNAs identified by TRAC‐seq. G) Quantitative comparison of m^7^G tRNA expression levels between Ctrl and cKO mice. H) Quantitative comparison of expression fold change between m^7^G and non‐m^7^G tRNAs. The expression of each tRNA type was calculated as mentioned above. I) Northern blot analysis of representative tRNAs in Ctrl and cKO mice. J) Polysome analysis of Ctrl and cKO mice: The polysome peaks in cKO mouse hepatocytes were significantly reduced compared to Ctrl mice. K) Quantification of the polysome‐to‐monosome ratio in Ctrl and cKO mice. L) The puromycin intake assay showed an increase in protein synthesis levels after PHx in Ctrl mice, whereas a decrease was observed with METTL1 knockout, and supplementation with METTL1 restored protein synthesis levels. β‐Actin was used as a loading control for Western blot analysis. U6 snoRNA was used as the loading control for Northwestern and Northern blotting analysis. Significance is indicated as follows: ^*^ *P*<0.05, ^**^ *P*<0.01, ^***^ *P*<0.001, and ns indicates no significant difference.

To explore the molecular mechanisms underlying METTL1‐mediated m^7^G tRNA modification in liver regeneration, we performed a Northwestern blot to assess m^7^G tRNA modification in the *Mettl1* cKO and WT mice. The results showed that depletion of METTL1 led to a significant decrease in tRNA m^7^G modification (**Figure** **4**A). This finding was further corroborated by liquid chromatography‐tandem mass spectrometry (LC‐MS/MS) (Figure 4B). We then applied the previously established m^7^G site‐specific tRNA reduction and cleavage sequencing (TRAC‐seq)^[^ ^24^, ^29^ ^]^ to study the m^7^G tRNA methylome in hepatocytes after PHx. Our TRAC‐seq data identified a total of 12 tRNAs with m^7^G modification in hepatocytes, predominantly enriched at the “RGGUY” motif (Figure 4C,D). Interestingly, the signals and expression levels of the identified m^7^G‐modified tRNAs were significantly decreased in the *Mettl1* cKO mice compared to the control group, whereas non‐m^7^G‐modified tRNAs remained largely unchanged (Figure 4E–H). Consistent with these findings, Northern blot analysis confirmed that METTL1 knockout specifically reduced the abundance of m^7^G‐modified tRNAs (ProAGG and ValAAC), but not that of an unmodified tRNA (GlnCTG) (Figure 4I). Our data indicated that the depletion of METTL1 led to decreased m^7^G modification and expression of target tRNAs, further confirming the vital function of METTL1‐mediated m^7^G tRNA modification in liver regeneration after PHx.

To elucidate the impact of m^7^G tRNA modification on mRNA translation during liver regeneration after PHx, we performed a polysome profiling assay. The results showed a decreased polyribosome peak and a reduced polysome‐to‐monosome ratio in *Mettl1* cKO mice, indicating a decrease in global mRNA translation upon METTL1 knockout (Figure 4J,K). Consistent with this finding, puromycin intake assays also demonstrated a significant decrease in nascent protein synthesis in the *Mettl1* cKO livers (Figure 4L). In summary, our findings highlight the critical role of METTL1‐mediated m^7^G tRNA modification in mRNA translation during liver regeneration after PHx.

### 2.5 METTL1 Affects the Translation Efficiency of YAP/TAZ in the Hippo Pathway

**Figure 5:**
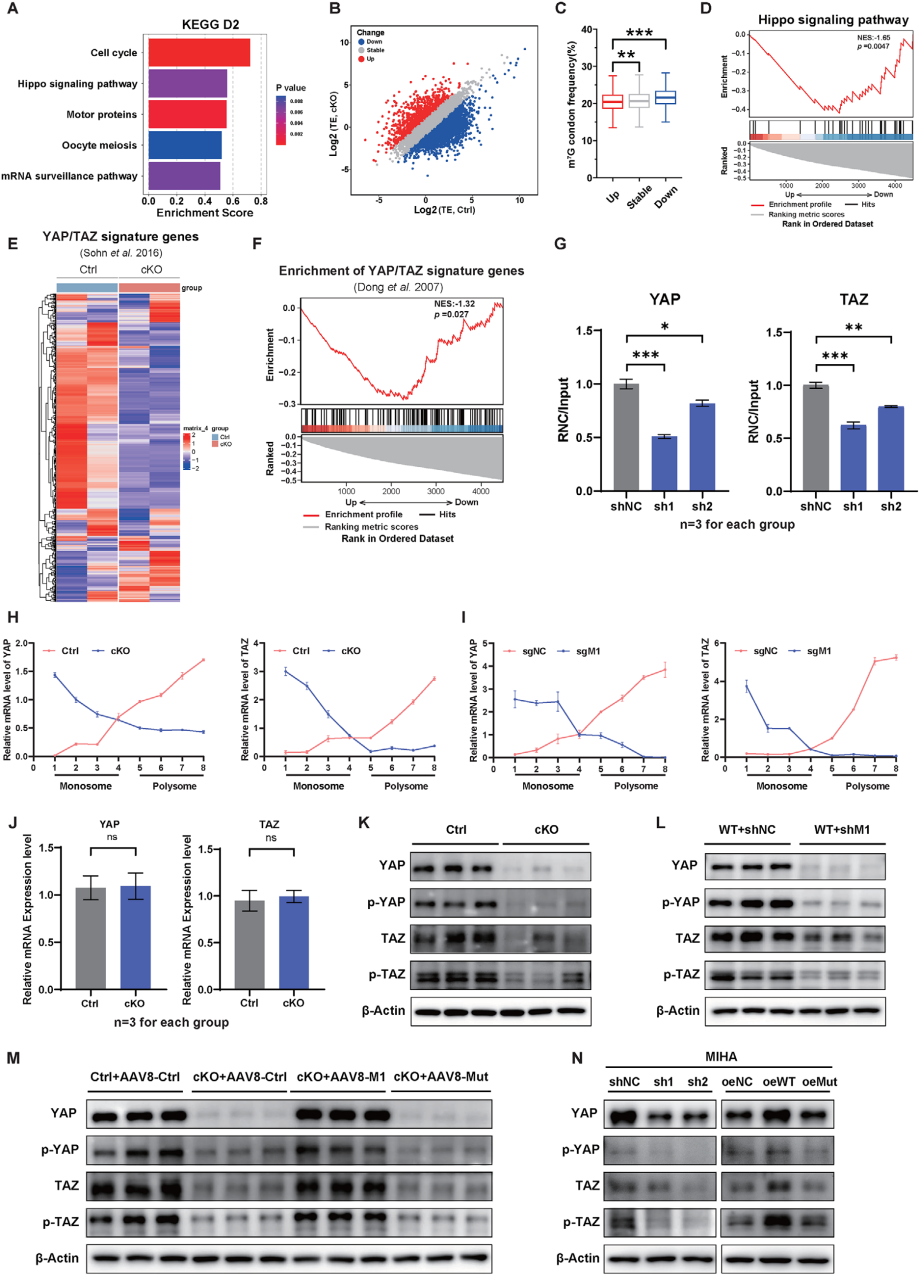
METTL1‐Mediated m^7^G Modification Regulates the Translation Efficiency of Key Hippo Pathway Proteins YAP/TAZ. A) Gene set enrichment analysis (GSEA) of proteomics data revealed that the Hippo signaling pathway was significantly enriched on D2 after PHx in Ctrl mice. B) The ribo‐seq scatter plot displayed changes in translation efficiency (TE) in the regenerating liver tissues of Ctrl and cKO mice on D2 after PHx. C) Frequency of m^7^G‐related codons in genes with altered TE. D) GSEA analysis of ribo‐seq data revealed that mRNAs with decreased TE in regenerating liver tissues of cKO mice on D2 after PHx were enriched for the Hippo signaling pathway. E) A heatmap based on Ribo‐seq data illustrated the TE of YAP/TAZ signature genes in regenerating liver tissues of Ctrl and cKO mice on D2 after PHx. F) GSEA analysis identified that mRNAs with decreased TE in regenerating liver tissues of cKO mice on D2 after PHx were enriched for YAP/TAZ signature genes. G) qRT‐PCR analysis of YAP/TAZ TE (RNC mRNA/Input mRNA) in normal hepatocytes (MIHA) with METTL1 knockdown (sh1, sh2) and control (shNC). H) Polysome profiling and qRT‐PCR analysis of YAP/TAZ mRNA levels in different sucrose gradient fractions in hepatocytes from Ctrl and cKO mice. I) Polysome profiling and qRT‐PCR analysis of YAP/TAZ mRNA levels in different sucrose gradient fractions in hepatocytes with METTL1 knockdown (sgM1) and control (sgNC). J) Relative mRNA expression levels of YAP and TAZ in Ctrl and cKO mice. K–M) Western blot analysis of key Hippo pathway proteins in regenerating liver tissues of various mouse models after PHx. K: Ctrl and cKO mice. L: Mice with METTL1 knockdown via AAV8. M: Mice with METTL1 overexpression via AAV8. Western blot analysis of key Hippo pathway proteins in normal hepatocytes (MIHA) with depletion or overexpression of METTL1. β‐Actin was used as a loading control for Western blot analysis. Significance is indicated as follows: ^*^ *P*<0.05, ^**^ *P*<0.01, ^***^ *P*<0.001, and ns indicates no significant difference.

Upon further investigation, we delved into the downstream mRNA targets affected by METTL1‐mediated m^7^G tRNA modification. Analysis of proteomic data identified a significant enrichment of the Hippo signaling pathway on the second day after PHx (**Figure** **5**A). Notably, LATS1/2 activity remained largely unchanged following PHx (Figure S5A, Supporting Information), suggesting the activation of a non‐canonical Hippo signaling mechanism during liver regeneration. Given the essential function of the Hippo pathway in tissue regeneration and organ enlargement,^[^ ^30^, ^31^, ^32^, ^33^ ^]^ we hypothesized that METTL1 might influence liver regeneration through the translation of the Hippo signaling pathway. To confirm this hypothesis, we performed ribosome sequencing (Ribo‐seq) to analyze the actively translating mRNAs (Figure 5B). Further analysis indicated that mRNAs with reduced TE had a notably higher frequency of codons decoded by m^7^G‐modified tRNAs (m^7^G‐related codons), suggesting that the m^7^G tRNA modification mediated by METTL1 played a vital role in regulating mRNA translation in a codon frequency‐dependent mechanism (Figure 5C). Furthermore, Gene Set Enrichment Analysis (GSEA) also revealed that mRNAs with TE down were enriched in the Hippo signaling pathway (Figure 5D). In addition, we also found that TE of the reported YAP/TAZ signature^[^ ^30^, ^34^ ^]^ was significantly decreased in the liver tissue of *Mettl1* cKO mice (Figure 5E,F). Notably, Ribo‐seq profiles of these genes showed strong concordance with the proteomic data (Figure S5B, Supporting Information), further implying that METTL1 regulated the translation of the YAP/TAZ in the Hippo signaling pathway.

We then conducted ribosome‐nascent chain‐complex‐bound mRNA (RNC) and polysome mRNA qPCR assay to further investigate the TE of YAP/TAZ. The results showed a significant decrease in the TE level of the YAP/TAZ in METTL1‐depleted hepatocytes (Figure 5G). Analysis of RNAs isolated from sucrose gradient fractionations also revealed decreased YAP/TAZ mRNA in actively translated polysome fractions in *Mettl1* cKO mice (Figure 5H) and METTL1‐depleted hepatocytes (Figure 5I), whereas total YAP/TAZ mRNA levels remained largely unaltered (Figure 5J). Correspondingly, the protein expression levels of YAP/TAZ and its phosphorylation levels were impaired with METTL1 depletion (Figure 5K,L). Moreover, overexpression of wild‐type METTL1, but not the catalytically inactive METTL1, increased the protein and its phosphorylation expression levels of YAP/TAZ in liver tissue and MIHA cells (Figure 5M,N).

To determine whether this translational regulation depends on tRNA m^7^G modification, we performed a tRNA complementation assay using tRNA‐ProAGG, a strongly downregulated METTL1 target with cognate codons enriched in the YAP/TAZ transcript. Expression of wild‐type tRNA‐ProAGG restored YAP/TAZ protein levels in METTL1‐knockdown cells, whereas an m^7^G‐deficient mutant (G46C) failed to do so (Figure S5C, Supporting Information), confirming the requirement of m^7^G modification for efficient YAP/TAZ translation. To further assess codon‐dependent regulation, we designed firefly luciferase reporters containing either the wild‐type YAP coding sequence or a synonymously mutated version. Under METTL1 knockdown, luciferase activity from the wild‐type reporter was significantly reduced, whereas the synonymously mutated reporter retained substantially higher translational activity (Figure S5D, Supporting Information). Together, these data suggest that the METTL1‐mediated m^7^G modification seletively promotes the translation of the Hippo pathway genes.

### 2.6 YAP is a Key METTL1 Target that Mediates Liver Regeneration After PHx

Given the known functional redundancy between YAP and TAZ in the Hippo pathway,^[^ ^35^, ^36^ ^]^ we performed siRNA‐mediated individual and dual knockdown of YAP and TAZ in mice following PHx. The results demonstrated that individual knockdown of either YAP or TAZ partially impaired liver regeneration, while dual knockdown caused a more severe defect (Figure S6A–C, Supporting Information), confirming that YAP and TAZ collectively contribute to METTL1‐dependent liver regeneration.

**Figure 6:**
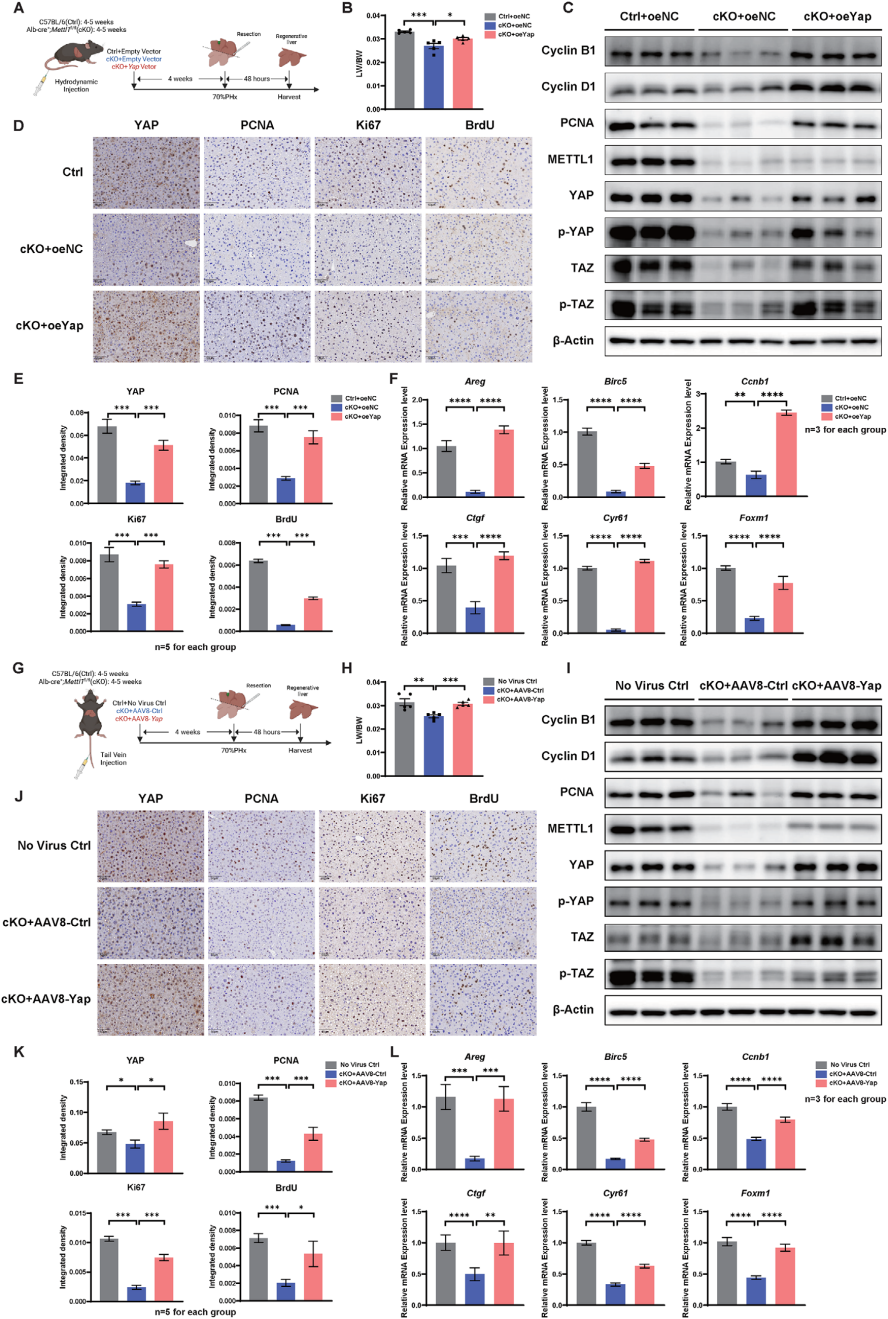
Overexpression of YAP in METTL1‐deficient Mice Restores Liver Regeneration after PHx. A) Experimental scheme: 4–5 weeks‐old C57BL/6 (Ctrl) and Alb‐cre^+^, Mettl1^fl/fl^ (cKO) mice were injected with YAP plasmid (cKO+oe*Yap*) or empty plasmid (cKO+oeNC) via hydrodynamic injection, and 70% PHx was performed 4 weeks later, followed by tissue collection 48 h after PHx. B,C) Ratio of LW to BW (B), protein expression level of proliferation‐related markers, and key proteins of the Hippo pathway (C) in Ctrl+oeNC, cKO+oeNC, and cKO+oe*Yap* mice at D2 after PHx. D–F) IHC staining and quantification of YAP, PCNA, Ki67, BrdU D,E), and relative mRNA expression levels of YAP downstream pro‐proliferative target genes (F) in regenerating liver tissues of the three groups on D2 after PHx. G) Experimental scheme: 4–5 weeks‐old C57BL/6 (Ctrl) and Alb‐cre^+^, Mettl1^fl/fl^ (cKO) mice were injected via tail vein with AAV8‐Ctrl or AAV8‐*Yap*, and 70% PHx was performed 4 weeks later, followed by tissue collection 48 h after PHx. H,I) Ratio of LW to BW (H), protein expression level of proliferation‐related markers, and key proteins of the Hippo pathway (I) in No Virus Ctrl, cKO+AAV8‐Ctrl, and cKO+AAV8‐*Yap* mice at D2 after PHx. J–L) IHC staining and quantification of YAP, PCNA, Ki67, BrdU (J‐K), and relative mRNA expression levels of YAP downstream pro‐proliferative target genes (L) in regenerating liver tissues of the three groups on D2 after PHx. β‐Actin was used as a loading control for Western blot analysis. Scale bars for IHC: 50 µm. Significance is indicated as follows: ^*^ *P*<0.05, ^**^ *P*<0.01, ^***^ *P*<0.001, ^****^ *P*<0.0001, and ns indicates no significant difference.

To further determine the function of YAP mediated by METTL1 in the regulation of liver regeneration after PHx, we performed rescue experiments by overexpressing *Yap* in *Mettl1* cKO mice via tail‐vein hydrodynamic injection (**Figure** **6**A). Successful *Yap* overexpression was confirmed by qRT‐PCR analysis (Figure S6D, Supporting Information). Our data showed that overexpression of *Yap* significantly promoted liver regeneration in *Mettl1* cKO mice, as evidenced by the liver weight/body weight ratio (Figure 6B). Consistently, upon overexpression of *Yap*, the expression levels of cell cycle markers were significantly upregulated, and cell proliferation, as indicated by the index scores of Ki67, PCNA, and BrdU, was also increased as expected (Figure 6C‐E). Furthermore, *Yap* overexpression was associated with an accelerated recovery of liver function (Figure S6E, Supporting Information). Additionally, we also utilized an AAV8 packaging *Yap* (AAV8‐*Yap*) model to promote *Yap* expression in *Mettl1* cKO mice (Figure 6G; Figure S6F, Supporting Information). Similarly, overexpression of *Yap* led to enhancement of liver regeneration, upregulation of cyclin proteins, and increased expression of proliferation markers, further supporting the role of YAP in promoting liver regeneration in *Mettl1* cKO mice (Figure 6H–K; Figure S6G, Supporting Information).

As previously reported, the Hippo pathway plays a crucial role in controlling organ growth, stem cell function, and tissue regeneration.^[^ ^33^, ^37^ ^]^ This pathway transmits signals from the plasma membrane to the nucleus and is involved in cellular processes such as proliferation, survival, and differentiation, by regulating the expression of various target genes, including *Areg, Birc5, Ctfg, Ccnb1, Cyr61, and Foxm1*.^[^ ^30^, ^38^ ^]^ By performing qRT‐PCR, we also found that overexpression of *Yap* significantly increased the mRNA levels of YAP‐activated genes compared to the control group mice (Figure 6F,L). Additionally, the mRNA level of these target genes was also upregulated after PHx and with overexpression of METTL1 (Figure S7A,D, Supporting Information), while notably decreased with the depletion of METTL1 (Figure S7B,C, Supporting Information). Taken together, these results suggested that YAP played a critical role in METTL1‐mediated liver regeneration after PHx.

### 2.7 Clinical Impact of METTL1 on Liver Regeneration after PHx

**Figure 7:**
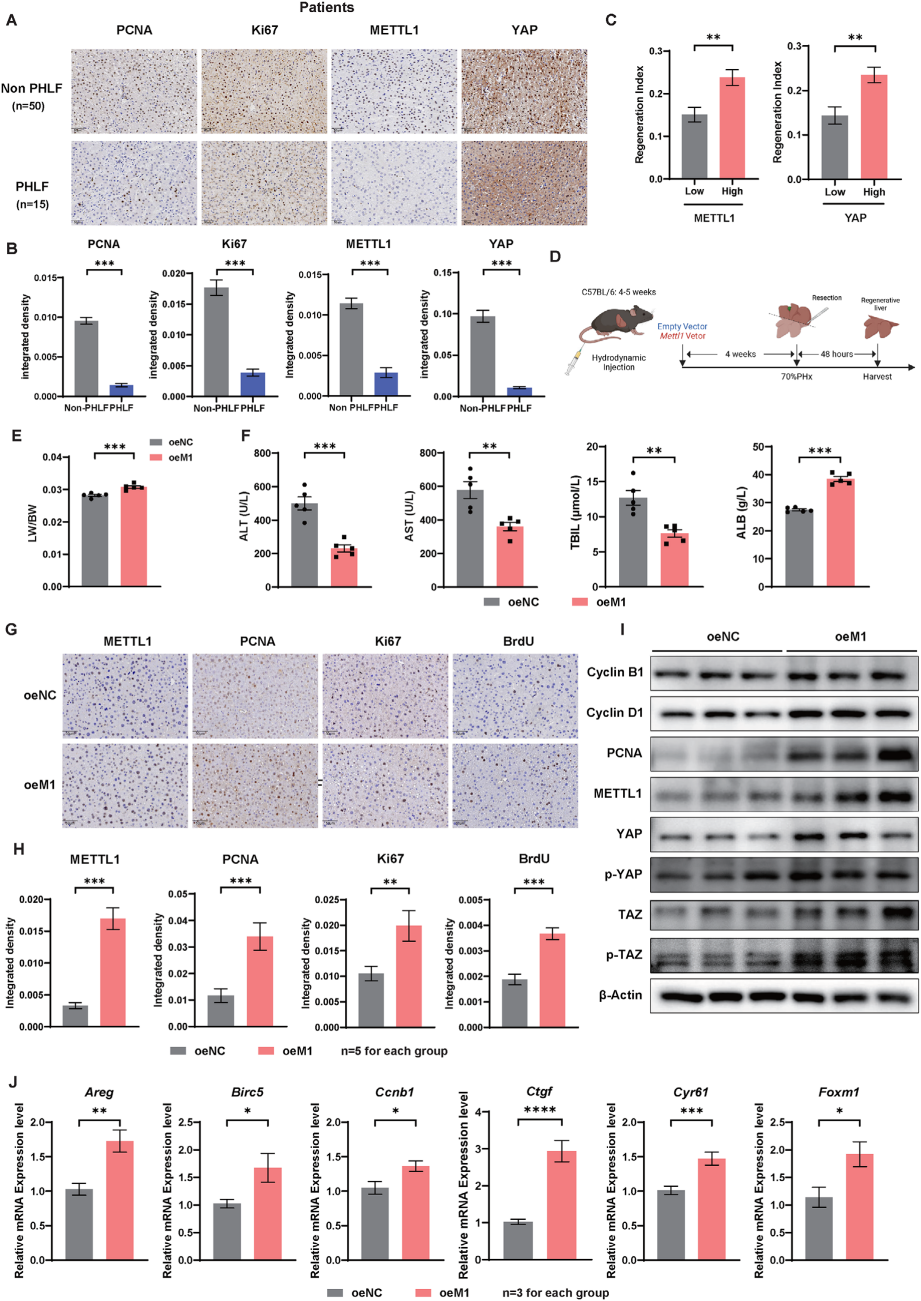
Potential Clinical Applications of METTL1 in Promoting Liver Regeneration. A,B) IHC staining (A) and statistical analysis (B) of PCNA, Ki67, METTL1, and YAP in normal liver tissues from patients in Non‐PHLF (n = 50) and PHLF (n = 15) groups. C) Correlation between METTL1 and YAP expression levels and liver volume regeneration rate after PHx. D) Experimental schematic: 4–5 weeks‐old C57BL/6 (Ctrl) mice were injected with either Mettl1 plasmid (Ctrl+oe*M1*) or empty vector plasmid (Ctrl+oeNC) via hydrodynamic injection, and 70% PHx was performed 4 weeks later, followed by tissue collection 48 h after PHx. E,F) Ratio of LW to BW (E) and serum levels of ALT/AST/TBIL/ALB (F) in Ctrl+oeNC and Ctrl+oeM1 mice at D2 after PHx. G–I) IHC staining (G,H) and Western blot (I) analysis of proliferation‐related markers and key proteins of the Hippo pathway in the above two groups. J) Relative mRNA expression levels of YAP downstream proliferation‐related target genes in the above two groups. β‐Actin was used as a loading control for Western blot analysis. Scale bars for IHC: 50 µm. Significance is indicated as follows: ^*^ *P*<0.05, ^**^ *P*<0.01, ^***^ *P*<0.001, and ns indicates no significant difference.

To further validate the clinical impact of METTL1 in liver regeneration after PHx, we performed IHC staining on normal liver tissues obtained from patients who underwent partial hepatectomy. The results indicated that the levels of proliferation‐related markers (PCNA, Ki67) in the non‐PHLF group (N = 50) were significantly higher than those in the PHLF group (N = 15). Additionally, the expression levels of METTL1 and YAP were also markedly higher in the non‐PHLF group (**Figure** **7**A,B). To further assess the clinical relevance of METTL1 and YAP in liver regeneration, we calculated the regeneration index (regenerated liver volume/remaining liver volume after hepatectomy) using 3D liver volume imaging software.^[^ ^39^ ^]^ The results revealed that patients with higher METTL1 and YAP expression levels exhibited a significantly higher regeneration index (Figure 7C). Conversely, neither METTL1/YAP expression nor the liver regeneration index correlated significantly with other clinical parameters, including preoperative liver function or intraoperative blood loss (Tables S1–S3, Supporting Information). This finding indicated that METTL1 and YAP played a crucial role in promoting the rapid proliferation of the residual liver after PHx.

Given the critical regulatory role of METTL1 in liver regeneration following PHx, we further investigated the potential clinical application of METTL1 in treating PHLF by conducting a METTL1 overexpression mouse model through hydrodynamic transfection (Figure 7D). As expected, the supplement of METTL1 resulted in a significant enhancement of liver regeneration after PHx compared to control groups, as evidenced by a marked increase in liver weight/body weight ratio on the second day after PHx (Figure 7E). Additionally, METTL1 effectively mitigated liver function damage (Figure 7F) and promoted the expression of cell cycle markers and proliferation‐related indicators (Figure 7G–I). Furthermore, replenishment of METTL1 also resulted in increased mRNA levels of YAP‐activated genes (Figure 7J). These findings suggested that METTL1 effectively promotes liver regeneration and improves liver function after PHx, offering a potential therapeutic target for the clinical treatment of PHLF.

## 3 Discussion

The liver exhibits remarkable regenerative capacity in response to toxin‐induced injury or surgical resection.^[^ ^9^, ^10^ ^]^ Liver homeostasis under stress conditions is maintained through the prompt regulation of critical gene expression.^[^ ^40^, ^41^, ^42^ ^]^ For example, studies have reported that the disruption of STAT3 signaling through the deletion of histone deacetylase 3 (HDAC3) or m^6^A methyltransferase Mettl14 impeded liver regeneration by hindering STAT3 nuclear translocation and diminishing the expression of m^6^A modification‐dependent proteins, culminating in substantial hepatocyte death and impaired liver regeneration.^[^ ^40^, ^41^ ^]^ However, the mechanisms of RNA modifications, as an essential component of epigenetic modifications, in liver regeneration remain unresolved. In this study, a combination of proteomics data, RNA sequencing, and Ribo‐seq was employed to elucidate alterations in RNA modification levels during liver regeneration. Our findings indicate a significant upregulation of METTL1 expression and its mediated tRNA m^7^G modification at various time points after hepatectomy, with the loss of METTL1 markedly impeding liver regeneration after hepatectomy, and identifying METTL1 as a critical target for promoting liver regeneration after hepatectomy. Additionally, our analysis of PHLF patients indicated a reduction in METTL1 expression in normal hepatocytes, correlating with a decreased rate of residual liver regeneration after hepatectomy (Figure S8, Supporting Information). These findings provide a theoretical foundation for the clinical utilization of METTL1 and its mediated tRNA m^7^G modification in promoting the regeneration and repair of residual liver tissue.

Through various animal models, including a liver‐specific conditional *Mettl1* knockout model, hydrodynamic injection, and AAV‐related mouse models, together with gain‐ and loss‐of‐function studies, we have elucidated METTL1's ability to restore the regeneration rate of residual liver tissue and improve liver function after hepatectomy, further underscoring the critical role of METTL1‐mediated m^7^G tRNA modification in liver regeneration. Mechanistically, proteomic and Ribo‐seq studies have revealed that METTL1‐mediated m^7^G tRNA modification selectively governs the translation level of the Hippo signaling pathway through a codon frequency‐dependent mechanism.

The Hippo signaling pathway, initially identified in Drosophila,^[^ ^30^ ^]^ is a crucial regulator of organ growth, stem cell function, and tissue regeneration.^[^ ^31^, ^43^ ^]^ The key effectors of this pathway, YAP/TAZ, were increasingly recognized as vital for organ regeneration. Their aberrant activation can lead to excessive growth (e.g., liver)^,[^ ^44^, ^45^ ^]^ induce dedifferentiation of mature cell types (e.g., hepatocytes),^[^ ^46^ ^]^ and stimulate stem cell amplification and differentiation.^[^ ^47^, ^48^ ^]^ For instance, Lin et al. demonstrated that inducing YAP overexpression in the myocardium of adult mice following myocardial infarction improved heart function and post‐infarction survival rate.^[^ ^49^ ^]^ Similarly, studies on liver regeneration have revealed that YAP protein levels increase after hepatectomy, with nuclear YAP expression correlating positively with hepatocyte proliferation.^[^ ^50^, ^51^ ^]^ Deletion of YAP in hepatocytes diminishes nuclear pSmad2 accumulation, suppresses epithelial‐mesenchymal transition (EMT)‐like responses, and inhibits proliferation.^[^ ^52^ ^]^ Interestingly, previous research indicated that YAP protein levels significantly increased during liver regeneration after hepatectomy, with its mRNA levels remaining unchanged, a finding corroborated in our present study (Figure S9, Supporting Information). This suggests that the regulation of YAP during liver regeneration might not occur at the traditional transcriptional level but rather post‐transcriptional or non‐transcriptional mechanisms.^[^ ^50^ ^]^ Our work further addresses this gap by uncovering a novel mechanism, which shows that METTL1‐mediated m^7^G tRNA selectively regulates the translation efficiency of YAP/TAZ during liver regeneration. This discovery identifies a potential therapeutic intervention at the translation level. Given the conserved role of YAP/TAZ as master regulators of growth across tissues^[^ ^44^, ^53^ ^]^ together with reports implicating METTL1 in promoting proliferation in cancers such as colorectal and lung cancer,^[^ ^54^, ^55^ ^]^ the METTL1‐m^7^G‐YAP axis likely represents a broader regulatory pathway operative in multiple tissues and pathological states. This functional conservation positions METTL1 as a promising common therapeutic target for a range of regenerative and proliferative disorders.

In our study, through the METTL1 overexpression model, we further demonstrated that METTL1 could effectively promote liver regeneration and improve liver function after hepatectomy, providing a foundation for potential clinical applications of METTL1 in treating PHLF. Although METTL1 has been implicated in hepatocellular carcinoma (HCC) progression, its oncogenic effects often involve cooperation with established cancer‐related pathways.^[^ ^19^, ^56^ ^]^ Importantly, our preliminary in vivo experiments showed that transient overexpression of METTL1 alone did not induce spontaneous tumor formation, suggesting that its tumor‐promoting potential may require additional signals from a permissive microenvironment. Recent advancements in synthetic and natural delivery methods, such as adenoviruses, retroviruses/lentiviruses, and adeno‐associated viruses (AAVs), have shown promise in delivering RNA and DNA molecules in vivo,^[^ ^57^, ^58^, ^59^ ^]^ indicating the feasibility of targeting METTL1 and its mediated m^7^G tRNA in clinical settings.

The limitations of our study should be noted. On one hand, it focused on hepatocytes, which represent the majority of liver cells.^[^ ^60^ ^]^ Although previous studies have emphasized the roles of bile duct cells and stromal cells in liver regeneration after hepatectomy, we found that METTL1 was barely detectable in these populations and therefore cannot characterize the translational regulation changes in these cell types. A comprehensive analysis of various cell types within regenerating liver tissues is essential to fully understand the cellular mechanisms of liver regeneration. On the other hand, consistent with previous reports indicating that hepatocyte proliferation peaks ≈48 h after PHx,^[^ ^21^, ^61^, ^62^ ^]^ our study also observed maximal proliferation on the second day post‐surgery and consequently focused on this time point for further analysis. However, it is essential to note that hepatocyte proliferation continues beyond the second day, with some cells still dividing up to the seventh day after hepatectomy. Therefore, investigating multiple time points after hepatectomy would facilitate a more comprehensive understanding of gene remodeling during liver regeneration.

In conclusion, we conducted a comprehensive translational profiling of regenerating liver after hepatectomy, elucidating the critical role of METTL1 in facilitating liver regeneration. The mechanistic investigations demonstrate that METTL1 promotes residual liver regeneration by regulating the translational efficiency of key genes YAP/TAZ within the Hippo pathway, consequently enhancing the transcription of downstream proliferation and cycle‐related target genes. These findings suggest that targeting METTL1 could present a promising therapeutic approach for preventing PHLF by promoting hepatocyte proliferation.

## 4 Experimental Section

### Animals

The C57BL/6 used in this study was purchased from JiCui Pharmaceutical Co., Ltd. (Guangdong, China). The genetically engineered mouse C57BL/6‐Mettl1^fl/fl^ was purchased from Beijing Biocytogen Co., Ltd. All mice used in this study, including Alb‐Cre^+^, Mettl1^fl/fl^ (cKO) mice and control mice Mettl1^fl/fl^ (Ctrl), were bred at the Experimental Animal Center of SYSU. The mice were randomly grouped into five/cages and housed in ventilated breeding facilities, with environmental control requirements maintaining a temperature of 20–26 °C (ideal temperature being 22 °C) and relative humidity at 40–60%. Air circulation was sufficient, with 10–15 air exchanges per hour, and positive pressure was maintained in the room to prevent external contamination. The light cycle was set to 12 h of light and 12 h of darkness, and noise levels were strictly controlled to avoid stress on the mice. The Institutional Ethics Committee approved all animal care and experimental protocols for Clinical Research and Animal Trials of SYSU‐FAH (*SYSU‐IACUC‐2023‐000183*). The study adhered to all relevant ethical regulations regarding the Reporting of In Vivo Experiments (ARRIVE) guidelines. Mice were euthanized according to institutional euthanasia criteria based on overall health condition. The ARRIVE1 reporting guidelines were used.^[^ ^63^ ^]^

### Statistical Analysis

All data were shown as mean ± SEM. Statistical analysis was performed using R software (Version 4.4.1) and GraphPad Prism 8.0 (GraphPad Software Inc., CA, USA). Statistical significance was determined using an unpaired two‐tailed Student's t‐test between two groups and one‐way ANOVA with Dunnett's post hoc test for multi‐group comparisons. Continuous variables were described as the median with IQR or the mean with SD, and Student's *t*‐test or the Mann‐Whitney *U* test was used to compare differences as appropriate. Categorical variables were described as numbers and percentages, and the *X^2^* analysis or *Fisher's* exact test was used to compare the differences between the two groups if necessary. All animal and cellular experiments were performed with at least three biological replicates (n ≥ 3). Correlations were assessed by the nonparametric Spearman's test. The p‐values were represented as follows: ^*^ *P*<0.05, ^**^ *P*<0.01, ^***^ *P*<0.001, ^∗∗∗∗^ *P*<0.0001, and not statistically significant when *P*>0.05.

## Conflict of Interest

The authors declare no conflict of interest.

## Author Contributions

M.H., S.L., Y.Z., and J.C. contributed equally to this work. SS, SL, MK, and YH designed the research and obtained funding. SS and SL served as guarantors for the overall content; MH, SL, YZ, DA, and ZH performed in vitro experiments. SL was responsible for the construction of the 70% PHx animal model, while SL, LH, ZS, JC, and XY were involved in grouping and implementing the animal experiments. SL, YZ, LH, and JC were responsible for tissue collection, and SL evaluated regenerated liver weight and analyzed the data. MH and YZ established the immortalized human liver (MIHA) cell line. MH, JC, YZ, and YL conducted ribosome‐related messenger RNA sequencing analysis, GSEA analysis, and correlation analysis. SS, SL, FW, and MK supervised the study, developed the protocol, and coordinated tissue collection. MH and SL drafted the manuscript, and all authors revised and approved the final manuscript.

## Supporting information



Supporting Information

Supporting Information

Supporting Information

Supporting Information

Supporting Information

Supporting Information

Supporting Information

Supporting Information

Supporting Information

Supporting Information

Supporting Information

## Data Availability

The data that support the findings of this study are available from the corresponding author upon reasonable request.

## References

[1] A. Sultana , M. Brooke‐Smith , S. Ullah , J. Figueras , M. Rees , J.‐N. Vauthey , C. Conrad , T. J. Hugh , O. J. Garden , S. T. Fan , M. Crawford , M. Makuuchi , Y. Yokoyama , M. Büchler , R. Padbury , HPB (Oxford) 2018, 20, 462. 29287736 10.1016/j.hpb.2017.11.007

[2] M. A. J. Van Den Broek , S. W. M. Olde Damink , C. H. C. Dejong , H. Lang , M. Malagó , R. Jalan , F. H. Saner , Liver Int 2008, 28, 767. 18647141 10.1111/j.1478-3231.2008.01777.x

[3] N. Sato , A. Kenjo , S. Suzushino , T. Kimura , R. Okada , T. Ishigame , Y. Kofunato , S. Marubashi , World J. Surg. 2021, 45, 3660. 34392399 10.1007/s00268-021-06289-9

[4] L. V. Saadat , B. C. Brajcich , Y. Liu , C. Ko , M. I. D'Angelica , HPB (Oxford) 2021, 23, 551. 32952033 10.1016/j.hpb.2020.08.013 PMC8422033

[5] F. F. Mohammed , C. J. Pennington , Z. Kassiri , J. S. Rubin , P. D. Soloway , U. Ruther , D. R. Edwards , R. Khokha , Hepatology 2005, 41, 857. 15726641 10.1002/hep.20618

[6] N. Fausto , J. S. Campbell , K. J. Riehle , Hepatology 2006, 43, S45. 16447274 10.1002/hep.20969

[7] R. Taub , Nat. Rev. Mol. Cell Biol. 2004, 5, 836. 15459664 10.1038/nrm1489

[8] G. K. Michalopoulos , Compr Physiol 2013, 3, 485. 23720294 10.1002/cphy.c120014

[9] L. Campana , H. Esser , M. Huch , S. Forbes , Nat. Rev. Mol. Cell Biol. 2021, 22, 608. 34079104 10.1038/s41580-021-00373-7

[10] G. K. Michalopoulos , M. C. DeFrances , Science 1997, 276, 60. 9082986 10.1126/science.276.5309.60

[11] E. A. Orellana , E. Siegal , R. I. Gregory , Nat. Rev. Genet. 2022, 23, 651. 35681060 10.1038/s41576-022-00501-9 PMC11170316

[12] M. Fu , J. Gu , M. Wang , J. Zhang , Y. Chen , P. Jiang , T. Zhu , X. Zhang , Mol Cancer 2023, 22, 30. 36782290 10.1186/s12943-023-01739-5 PMC9926655

[13] L. Malbec , T. Zhang , Y.‐S. Chen , Y. Zhang , B.‐F. Sun , B.‐Y. Shi , Y.‐L. Zhao , Y. Yang , Y.‐G. Yang , Cell Res. 2019, 29, 927. 31520064 10.1038/s41422-019-0230-z PMC6889513

[14] V. Marchand , L. Ayadi , F. G. M. Ernst , J. Hertler , V. Bourguignon‐Igel , A. Galvanin , A. Kotter , M. Helm , D. L. J. Lafontaine , Y. Motorin , Angew Chem Int Ed Engl 2018, 57, 16785. 30370969 10.1002/anie.201810946

[15] Y. Liu , C. Yang , Y. Zhao , Q. Chi , Z. Wang , B. Sun , Aging (Albany NY) 2019, 11, 12328. 31866582 10.18632/aging.102575 PMC6949057

[16] P. Xia , H. Zhang , K. Xu , X. Jiang , M. Gao , G. Wang , Y. Liu , Y. Yao , X. Chen , W. Ma , Z. Zhang , Y. Yuan , Cell Death Dis. 2021, 12, 691. 34244479 10.1038/s41419-021-03973-5 PMC8270967

[17] Z. Dai , H. Liu , J. Liao , C. Huang , X. Ren , W. Zhu , S. Zhu , B. Peng , S. Li , J. Lai , L. Liang , L. Xu , S. Peng , S. Lin , M. Kuang , Mol. Cell 2021, 81, 3338. 10.1016/j.molcel.2021.07.003 34352206

[18] J. Liao , Y. Yi , X. Yue , X. Wu , M. Zhu , Y. Chen , S. Peng , M. Kuang , S. Lin , Z. Peng , Hepatology 2023, 77, 1896. 35698894 10.1002/hep.32615

[19] S. Zhu , Y. Wu , X. Zhang , S. Peng , H. Xiao , S. Chen , L. Xu , T. Su , M. Kuang , Mol. Ther. 2023, 31, 1596. 35965412 10.1016/j.ymthe.2022.08.004 PMC10278047

[20] S. Boyce , D. Harrison , Lab Anim (NY) 2008, 37, 529. 18948993 10.1038/laban1108-529

[21] Y. Liang , Q. Mei , E. He , P. Ballar , C. Wei , Y. Wang , Y. Dong , J. Zhou , X. Tao , W. Qu , M. Zhao , G. Chhetri , L. Wei , J. Shao , Y. Shen , J. Liu , L. Feng , Y. Shen , Cell Death Dis. 2024, 15, 681. 39289348 10.1038/s41419-024-07069-8 PMC11408687

[22] Y. Chen , L. Chen , X. Wu , Y. Zhao , Y. Wang , D. Jiang , X. Liu , T. Zhou , S. Li , Y. Wei , Y. Liu , C. Hu , B. Zhou , J. Qin , H. Ying , Q. Ding , Nat. Commun. 2023, 14, 1521. 36934083 10.1038/s41467-023-37247-9 PMC10024732

[23] M. Huang , J. Jiao , H. Cai , Y. Zhang , Y. Xia , J. Lin , Z. Shang , Y. Qian , F. Wang , H. Wu , X. Kong , J. Gu , Hepatology 2022, 76, 1706. 35288960 10.1002/hep.32458 PMC9790589

[24] S. Lin , Q. Liu , V. S. Lelyveld , J. Choe , J. W. Szostak , R. I. Gregory , Mol. Cell 2018, 71, 244. 29983320 10.1016/j.molcel.2018.06.001 PMC6086580

[25] Y. Huang , J. Ma , C. Yang , P. Wei , M. Yang , H. Han , H. D. Chen , T. Yue , S. Xiao , X. Chen , Z. Li , Y. Tang , J. Luo , S. Lin , L. Huang , Biomark Res 2022, 10, 68. 36071474 10.1186/s40364-022-00414-z PMC9454133

[26] H. Liu , X. Zeng , X. Ren , Y. Zhang , M. Huang , L. Tan , Z. Dai , J. Lai , W. Xie , Z. Chen , S. Peng , L. Xu , S. Chen , S. Shen , M. Kuang , S. Lin , Gut 2023, 72, 1555. 36283801 10.1136/gutjnl-2022-327230

[27] A. Rossi , R. Romano , S. Fecarotta , M. Dell'Anno , V. Pecorella , R. Passeggio , S. Zancan , G. Parenti , F. Santamaria , F. Borgia , F. Deodato , S. Funghini , C. A. Rupar , C. Prasad , M. O'Callaghan , J. J. Mitchell , M. G. Valsecchi , G. la Marca , S. Galimberti , A. Auricchio , N. Brunetti‐Pierri , Med 2025, 6, 100544. 39547230 10.1016/j.medj.2024.10.021

[28] N. Zabaleta , C. Unzu , N. D. Weber , G. Gonzalez‐Aseguinolaza , Nat. Rev. Gastroenterol. Hepatol. 2023, 20, 288. 36646909 10.1038/s41575-022-00729-0

[29] S. Lin , Q. Liu , Y. Z. Jiang , R. I. Gregory , Nat. Protoc. 2019, 14, 3220. 31619810 10.1038/s41596-019-0226-7 PMC8959837

[30] J. Dong , G. Feldmann , J. Huang , S. Wu , N. Zhang , S. A. Comerford , M. F. Gayyed , R. A. Anders , A. Maitra , D. Pan , Cell 2007, 130, 1120. 17889654 10.1016/j.cell.2007.07.019 PMC2666353

[31] A. Ramos , F. D. Camargo , Trends Cell Biol. 2012, 22, 339. 22658639 10.1016/j.tcb.2012.04.006 PMC3383919

[32] K. Namoto , C. Baader , V. Orsini , A. Landshammer , E. Breuer , K. T. Dinh , R. Ungricht , M. Pikiolek , S. Laurent , B. Lu , A. Aebi , K. Schönberger , E. Vangrevelinghe , O. Evrova , T. Sun , S. Annunziato , J. Lachal , E. Redmond , L. Wang , K. Wetzel , P. Capodieci , J. Turner , G. Schutzius , V. Unterreiner , M. Trunzer , N. Buschmann , D. Behnke , R. Machauer , C. Scheufler , C. N. Parker , et al., Cell Stem Cell 2024, 554–569, 517. 10.1016/j.stem.2024.03.003 38579685

[33] Z. Meng , F.‐L. Li , C. Fang , B. Yeoman , Y. Qiu , Y. Wang , X. Cai , K. C. Lin , D. Yang , M. Luo , V. Fu , X. Ma , Y. Diao , F. G. Giancotti , B. Ren , A. J. Engler , K.‐L. Guan , Sci. Adv. 2022, 8, abl9806. 10.1126/sciadv.abl9806 PMC9132450 35613278

[34] B. H. Sohn , J.‐J. Shim , S.‐B. Kim , K. Y. Jang , S. M. Kim , J. H. Kim , J. E. Hwang , H.‐J. Jang , H.‐S. Lee , S.‐C. Kim , W. Jeong , S. S. Kim , E. S. Park , J. Heo , Y. J. Kim , D.‐G. Kim , S.‐H. Leem , A. Kaseb , M. M. Hassan , M. Cha , I.‐S. Chu , R. L. Johnson , Y.‐Y. Park , J.‐S. Lee , Clin. Cancer Res. 2016, 22, 1256. 26459179 10.1158/1078-0432.CCR-15-1447 PMC5536176

[35] S. Ma , Z. Meng , R. Chen , K. L. Guan , Annu. Rev. Biochem. 2019, 88, 577. 30566373 10.1146/annurev-biochem-013118-111829

[36] Z. Meng , T. Moroishi , K. L. Guan , Genes Dev. 2016, 30, 1. 26728553 10.1101/gad.274027.115 PMC4701972

[37] J. H. Driskill , D. Pan , Nat. Rev. Mol. Cell Biol. 2023, 24, 895. 37626124 10.1038/s41580-023-00644-5

[38] T. Mizuno , H. Murakami , M. Fujii , F. Ishiguro , I. Tanaka , Y. Kondo , S. Akatsuka , S. Toyokuni , K. Yokoi , H. Osada , Y. Sekido , Oncogene 2012, 31, 5117. 22286761 10.1038/onc.2012.5

[39] S. Chen , S. Feng , J. Wei , F. Liu , B. Li , X. Li , Y. Hou , D. Gu , M. Tang , H. Xiao , Y. Jia , S. Peng , J. Tian , M. Kuang , Eur Radiol 2019, 29, 4177. 30666445 10.1007/s00330-018-5986-x

[40] X.‐F. Lu , X.‐Y. Cao , Y.‐J. Zhu , Z.‐R. Wu , X. Zhuang , M.‐Y. Shao , Q. Xu , Y.‐J. Zhou , H.‐J. Ji , Q.‐R. Lu , Y.‐J. Shi , Y. Zeng , H. Bu , Cell Death Dis. 2018, 9, 398. 29540666 10.1038/s41419-018-0428-x PMC5852132

[41] X. Cao , Y. Shu , Y. Chen , Q. Xu , G. Guo , Z. Wu , M. Shao , Y. Zhou , M. Chen , Y. Gong , C. Li , Y. Shi , H. Bu , Cell Mol. Gastroenterol. Hepatol. 2021, 12, 633. 33848642 10.1016/j.jcmgh.2021.04.001 PMC8261664

[42] S. Wang , C. Zhang , D. Hasson , A. Desai , S. SenBanerjee , E. Magnani , C. Ukomadu , A. Lujambio , E. Bernstein , K. C. Sadler , Dev. Cell 2019, 50, 43. 31231040 10.1016/j.devcel.2019.05.034 PMC6615735

[43] F. X. Yu , K. L. Guan , Genes Dev. 2013, 27, 355. 23431053 10.1101/gad.210773.112 PMC3589553

[44] I. M. Moya , G. Halder , Nat. Rev. Mol. Cell Biol. 2019, 20, 211. 30546055 10.1038/s41580-018-0086-y

[45] G. Loforese , T. Malinka , A. Keogh , F. Baier , C. Simillion , M. Montani , T. D. Halazonetis , D. Candinas , D. Stroka , EMBO Mol. Med. 2017, 9, 46. 27940445 10.15252/emmm.201506089 PMC5210079

[46] O. Alder , R. Cullum , S. Lee , A. C. Kan , W. Wei , Y. Yi , V. C. Garside , M. Bilenky , M. Griffith , A. S. Morrissy , G. A. Robertson , N. Thiessen , Y. Zhao , Q. Chen , D. Pan , S. J. M. Jones , M. A. Marra , P. A. Hoodless , Cell Rep. 2014, 9, 261. 25263553 10.1016/j.celrep.2014.08.046 PMC4612615

[47] L. Zheng , C. Xiang , X. Li , Q. Guo , L. Gao , H. Ni , Y. Xia , T. Xi , J. Hematol. Oncol. 2018, 11, 72. 29848346 10.1186/s13045-018-0613-5 PMC5977742

[48] J. K.‐H. Hu , W. Du , S. J. Shelton , M. C. Oldham , C. M. DiPersio , O. D. Klein , Cell Stem Cell 2017, 21, 91. 28457749 10.1016/j.stem.2017.03.023 PMC5501749

[49] Z. Lin , A. von Gise , P. Zhou , F. Gu , Q. Ma , J. Jiang , A. L. Yau , J. N. Buck , K. A. Gouin , P. R. R. van Gorp , B. Zhou , J. Chen , J. G. Seidman , D.‐Z. Wang , W. T. Pu , Circ. Res. 2014, 115, 354. 24833660 10.1161/CIRCRESAHA.115.303632 PMC4104149

[50] J. Dong , Mol. Med. Rep. 2012, 5, 410. 22012126 10.3892/mmr.2011.640

[51] J. L. Grijalva , M. Huizenga , K. Mueller , S. Rodriguez , J. Brazzo , F. Camargo , G. Sadri‐Vakili , K. Vakili , Am J. Physiol. Gastrointest Liver Physiol. 2014, 307, G196. 24875096 10.1152/ajpgi.00077.2014

[52] S. H. Oh , M. Swiderska‐Syn , M. L. Jewell , R. T. Premont , A. M. Diehl , J. Hepatol. 2018, 69, 359. 29758331 10.1016/j.jhep.2018.05.008 PMC6349217

[53] M. Maugeri‐Sacca , R. De Maria , Pharmacol. Ther. 2018, 186, 60. 29305295 10.1016/j.pharmthera.2017.12.011

[54] Z. Sun , Y. Xu , C. Si , X. Wu , Y. Guo , C. Chen , C. Wang , Mol. Cancer 2024, 23, 179. 39215345 10.1186/s12943-024-02090-z PMC11363613

[55] J. Ma , H. Han , Y. Huang , C. Yang , S. Zheng , T. Cai , J. Bi , X. Huang , R. Liu , L. Huang , Y. Luo , W. Li , S. Lin , Mol. Ther. 2021, 29, 3435. 10.1016/j.ymthe.2021.08.005 PMC8636169 34371184

[56] X. Zeng , G. Liao , S. Li , H. Liu , X. Zhao , S. Li , K. Lei , S. Zhu , Z. Chen , Y. Zhao , X. Ren , T. Su , A. S.‐L. Cheng , S. Peng , S. Lin , J. Wang , S. Chen , M. Kuang , Hepatology 2023, 25, 1122. 10.1002/hep.32585 35598182

[57] A. M. Keeler , T. R. Flotte , Annu. Rev. Virol. 2019, 6, 601. 31283441 10.1146/annurev-virology-092818-015530 PMC7123914

[58] J. J. Darrow , Drug Discov Today 2019, 24, 949. 30711576 10.1016/j.drudis.2019.01.019

[59] Z. Wang , H. Wang , S. Zhou , J. Mao , Z. Zhan , S. Duan , MEDCOMM – Oncol. 2024, 3, 93.

[60] G. K. Michalopoulos , B. Bhushan , Nat. Rev. Gastroenterol. Hepatol. 2021, 18, 40. 32764740 10.1038/s41575-020-0342-4

[61] S. Fan , Y. Gao , A. Qu , Y. Jiang , H. Li , G. Xie , X. Yao , X. Yang , S. Zhu , T. Yagai , J. Tian , R. Wang , F. J. Gonzalez , M. Huang , H. Bi , Hepatology 2022, 73, 74. 10.1002/hep.32105 PMC10259205 34387904

[62] Y. Gao , S. Fan , H. Li , Y. Jiang , X. Yao , S. Zhu , X. Yang , R. Wang , J. Tian , F. J. Gonzalez , M. Huang , H. Bi , Acta Pharm. Sin. B 2021, 11, 727. 33777678 10.1016/j.apsb.2020.11.021 PMC7982502

[63] N. Percie du Sert , V. Hurst , A. Ahluwalia , S. Alam , M. T. Avey , M. Baker , W. J. Browne , A. Clark , I. C. Cuthill , U. Dirnagl , M. Emerson , P. Garner , S. T. Holgate , D. W. Howells , N. A. Karp , S. E. Lazic , K. Lidster , C. J. MacCallum , M. Macleod , E. J. Pearl , O. H. Petersen , F. Rawle , P. Reynolds , K. Rooney , E. S. Sena , S. D. Silberberg , T. Steckler , H. Würbel , PLoS Biol. 2020, 18, 3000410. 10.1371/journal.pbio.3000410 PMC7360023 32663219

