# Supplementary material for: METTL1‐Mediated M7G tRNA Modification Promotes Residual Liver Regeneration After Hepatectomy via Translational Control
Source: Adv Sci (Weinh). 2025 Dec 8;13(12):e07329. doi: 10.1002/advs.202507329 (PMC12948282; doi:10.1002/advs.202507329)
Supplement: Supplementary file 1 — Supporting Information [file ADVS-13-e07329-s011.docx]

**Supplemental Methods and Materials**

**Supplementary Methods**

**Generation of liver-specific conditional Mettl1 knockout mice**

*Mettl1* conditional knockout (*Mettl1* cKO) mice were generated via crossing male Alb-Cre^+^ mice with female Mettl1^fl/wt^ mice. The genotype of transgenic mice was determined by amplifying target DNA using PCR and then analyzing it through agarose gel electrophoresis. The differences in the livers of Alb-Cre^+^, Mettl1^fl/fl^ mice, and wild-type mice were compared by liver weight/body weight and serum analysis.

**Partial hepatectomy model and sample collection**

Male mice between 8-10 weeks were anesthetized via isoflurane, and a midline laparotomy was performed to expose the surgical field. The left and median lobes of the liver were mobilized and delivered after ligating with a single silk 4-0 suture, following this, the left and median lobes were resected. The sham procedure involved only a midline laparotomy. For BrdU assays, mice were injected I.P. at 100 mg/kg in 0.9% saline two hours prior to sacrifice to label the proliferating cells. Serum and liver tissues were collected for further analysis and BrdU-positive cells were detected by immunohistochemistry staining.

**Clinical patients**

The data of HCC patients who underwent partial hepatectomy at the First Affiliated Hospital of Sun Yat-sen University (SYSU-FAH) between 2022 and 2024 were included and analyzed. The inclusion criteria were (1) patients who underwent hemi-hepatectomy, (2) detailed radiological data available for both the preoperative period and one month postoperatively. The exclusion criteria were (1) patients who underwent an irregular hepatectomy, (2) lack of preoperative or postoperative radiological data, (3) unable to calculate the patient's estimated liver resection volume or future liver volume. A total of 65 patients who underwent partial hepatectomy were included. This study was approved by The Institutional Review Board of SYSU-FAH (Ethical approval number: *LunShen [2022] No. 003*) and conducted following the Declaration of Helsinki. Perioperative data, including detailed clinical examination, laboratory evaluation (complete blood count, liver functions, kidney functions and alpha fetal protein (AFP), and radiological evaluation (abdominal ultrasonography and triphasic computed tomography and/or magnetic resonance imaging) were routinely performed. The characteristics of patients were shown in Table S4.

**Definitions of PHLF**

PHLF was characterized by abnormal serum bilirubin and INR levels on or after postoperative day 5 as defined by the International Study Group of Liver Surgery (ISGLS) ^[1]^.

**Volumetric analysis of the liver**

The contrast-enhanced CT or MRI scanning data in DICOM format for all patients were imported into the digital medical liver three-dimensional (3D) reconstruction system (YORKTAL). The software automatically recognized the liver parenchyma, the intrahepatic liver lesions, and vascular structures. 3D reconstruction was performed automatically, which could be modified manually when necessary. Based on the reconstructed 3D liver model, panel or serial lines were used to divide the liver. The total liver volume (TLV, ml), and future liver volume (FLV, ml) were automatically calculated by the software. The FLV ratio is defined as FLV/TLV to represent the percentage of liver remnant after resection.

Volume regeneration index (RI), defined as the regenerated liver volume one month after hepatectomy divided by the total liver volume before hepatectomy, was used as the main parameter to measure the level of regeneration in our study.

**Adeno-associated virus (AAV) tail vein injection model**

To evaluate the effects of METTL1 and YAP on liver regeneration after PHx, we established a model of hepatocyte-specific METTL1 knockdown in C57BL/6 mice (4-5 weeks, male) by administering hepatotropic adeno-associated virus serotype 8 (AAV8) that carries a short fragment for specific knockdown of Mettl1 (HBAAV8-m-Mettl1-shRNA-EGFP, 1.0 × 10^12^ viral genomes/mL) (AAV8-sh*Mettl1*) via tail vein injection, and the mice in the control group were administered with AAV8 that carries a negative control scrambled RNA fragment (HBAAV8-NC-shRNA-EGFP, 1.0 × 10^12^ viral genomes/mL) (AAV8-sh*NC*); The sequences of shRNA primers are shown in Table S5. We also established models of hepatocyte-specific METTL1 and YAP overexpression in Alb-Cre^+^, Mettl1^fl/fl^ (*Mettl1* cKO) mice (4-5 weeks, male) by administering AAV8 that carries a specific plasmid for overexpressing Mettl1 ([NM_010792.1](https://www.ncbi.nlm.nih.gov/nuccore/NM_010792.1)) (HBAAV8-TBG-m-Mettl1-3xflag-ZsGreen, 1.0 × 10^12^ viral genomes/mL) (AAV8-*Mettl1*), Mettl1 mut (HBAAV8-TBG-m-Mettl1-mut-3xflag-ZsGreen, 1.0 × 10^12^ viral genomes/mL) (AAV8-*Mut*) and Yap ([NM_001171147.1](https://www.ncbi.nlm.nih.gov/nuccore/NM_001171147.1)) (HBAAV8-TBG-m-Yap-3xflag-ZsGreen, 1.0 × 10^12^ viral genomes/mL) (AAV8-*Yap*) via tail vein injection, and the mice in the control group were administered with AAV8 that carries a negative control plasmid (HBAAV8-TBG-NC-ZsGreen, 1.0 × 10^12^ viral genomes/mL) (AAV8-Ctrl). All these constructs we used were purchased from Hanbio Biotechnology, Shanghai, China. Four weeks later, mice were anaesthetized to observe the accumulation of AAV8 in the liver via an In Vivo Imaging System (IVIS). After verifying the AAV efficiency, partial hepatectomy was performed in these mice, and serum and liver specimens were collected for subsequent experiments.

**Hydrodynamic tail-vein injection (HDTVi) mice model**

For HDTVi, groups of 4-5 weeks-old C57BL/6 and Alb-Cre^+^, Mettl1^fl/fl^ (*Mettl1* cKO) mice were used and the procedure was performed as described previously ^[2]^. A total of 30μg Mettl1 ([NM_010792.1](https://www.ncbi.nlm.nih.gov/nuccore/NM_010792.1)) overexpression plasmid (oe*Mettl1*), 30μg Yap ([NM_001171147.1](https://www.ncbi.nlm.nih.gov/nuccore/NM_001171147.1)) overexpression plasmid (oe*Yap*), or 30μg empty vector plasmid (oe*NC*) were diluted in 2ml saline, constituted a total volume of 10% of the mouse body weight, and administered via injection into the lateral tail vein of C57BL/6 and *Mettl1* cKO mice within 5 to 8 seconds. The mice received partial hepatectomy four weeks later, and serum and liver tissues were harvested for analysis.

**Liver animal imaging**

We conducted noninvasive multispectral fluorescence imaging *in vivo* using the In Vivo Imaging System (IVIS) (Perkinelmer, Waltham, Mass). Mice were anesthetized with 1-2% isoflurane (Attane, MINRAD Inc.) in O_2_ at a flow rate of 3-5 L/min, and a midline laparotomy was performed to expose the liver. The fluorescence intensity (eGFP) in the livers in each group was analyzed using the Ex.488/Em.509 filter channels. The accumulation of AAV8 in the liver was observed by quantifying the regions of interest in the displayed images using Living Image software 4.5.5 (Perkinelmer, Waltham, Mass), and the data were presented as average radiance (photons/sec/cm^2^/steradian).

**Biochemical assessment**

The levels of serum alanine aminotransferase (ALT), aspartate transaminase (AST), albumin (ALB) and total bilirubin (TBIL) were measured by commercially available kits (Wuhan Servicebio Biological Technology Co., LTD., China) following the manufacturer’s standard protocols.

**Northern Blot, Northwestern Blot and Western Blot**

The Northern blot, Northwestern blot and Western blot were performed as previously described ^[3-4]^. In brief, total protein was extracted using RIPA Lysis Buffer (Epizyme, PC101), phosphatase inhibitor cocktail (Thermo Fisher Scientific) and quantified with a commercial BCA Protein Assay Kit (Epizyme, ZJ101). 20 μg of total protein from each sample were loaded and separated by SDS-PAGE and then transferred from the gel onto a polyvinylidene fluoride (PVDF) membrane. After blocking, membranes were incubated overnight with corresponding primary antibodies at 4°C, followed by incubation with REAL EnVision-HRP antibodies (Agilent Technology, K5007). Finally, the Omni-ECL™ ultra-sensitive chemiluminescence detection kit was used for exposure to visualize signals.

For the Northern and Northwestern blot, total RNA was extracted using TRIzol (Thermo Fisher Scientific) following its instructions. A 15% TBE-UREA gel electrophoresis was used to separate 2 μg of total RNA samples, then transferred onto positively charged nylon membranes (Thermo Fisher Scientific). After ultraviolet (UV) crosslinking and blotted with digoxigenin-labeled probe specific to U6 snRNA. For Northwestern blotting of tRNA, after transferring and UV crosslinking, the membrane containing tRNA was blotted overnight at 4°C with an anti-m^7^G antibody under shaking conditions. Finally, the digoxigenin or anti-m^7^G antibody (MBL International, RN017M) signal was detected according to the previously described protein blotting protocol ^[4]^. The antibodies used are shown in Table S6.

**Proteomics**

The liver tissues after hepatectomy were collected for protein extraction by centrifugation. Then, a 4D label-free quantitative proteomics analysis was performed, including trypsin digestion, LC/MS-MS analysis, and data analysis. Jingjie PTM BioLabs supported the proteomics analysis. The raw sequencing data underwent processing through the MaxQuant search engine (v.1.6.15.0). Tandem mass spectra were matched against the Rat UniProt databases and concatenated with the reverse decoy database. Proteomics analysis was repeated twice per group. Data were normalized using the median centering method across total proteins to correct sample loading differences. Differential proteins were defined with a fold change ≥2 between every two groups. Using the Robust Rank Aggregation (RRA) method ^[5]^, proteins with a score ≤0.05 were identified as consistently upregulated proteins at every time point after hepatectomy. Furthermore, the resulting proteins with a lower coefficient of variation (CV) of the expression at each time point after hepatectomy were considered to be more stably upregulated. The ratio of protein expression of each sample at each time point after hepatectomy to the average expression of samples before hepatectomy was used to generate a heatmap. Spearman's rank correlation coefficient was applied to evaluate the associations between protein expression and TE.

**RNA sequencing**

Total RNA was extracted from PHx-induced regenerating liver tissue samples and subjected to transcriptome sequencing by Gene Denovo Biotechnology Co. (Guangzhou, China) according to the manufacturer's protocol. First, mRNA was enriched using oligo (dT) magnetic beads. Then, cDNA libraries were constructed using the NEBNext Ultra RNA Library Prep Kit, and paired-end sequencing (150 bp) was performed on the Illumina NovaSeq 6000 platform. The resulting data were quality controlled and filtered using FastQC and Cutadapt, aligned to the mouse reference genome using HISAT2, and quantified using featureCounts. The gene expression level was normalized by using the RPKM (Reads Per Kilobase per Million mapped reads) method. Differential genes were defined with a fold change ≥2 between every two groups.

**Immunohistochemistry (IHC)**

The liver tissues were fixed in 10% neutral buffered formalin for 24 hours and then sectioned at a thickness of 4 μm. The prepared sections were incubated at 65°C for 2 hours to facilitate dewaxing, followed by xylene immersion and rehydration through a graded ethanol series. After rehydration, antigen retrieval was performed using 1× EDTA buffer under high pressure. To inhibit endogenous peroxidase activity, the sections were incubated in 3% hydrogen peroxide solution at room temperature in the dark for 10 minutes, and non-specific binding sites were blocked with 20% goat serum for 30 minutes. Then, the sections were further incubated with primary antibodies overnight at 4°C and secondary antibodies for 30 minutes the next day. Color development was achieved using diaminobenzidine (DAB) as the chromogen, followed by counterstaining with hematoxylin. After staining, sections were dehydrated through an ascending ethanol series, cleared in xylene, and allowed to air dry. The sections were then mounted using neutral resin. Microscopic examination and image capture were conducted to document the results. The antibodies used are shown in Table S7.

**Immunofluorescence Staining**

Immunofluorescence staining was performed on paraffin-embedded tissue sections. Briefly, sections were baked at 65 °C for 3 h, deparaffinized in xylene, and rehydrated through graded ethanol to water. Antigen retrieval was carried out in EDTA buffer (pH 8.0) using a pressure cooker, followed by cooling to room temperature. After permeabilization with 0.5% Triton X-100, sections were blocked with 10% goat serum for 30 min at room temperature and incubated with primary antibodies (diluted in 1% goat serum) overnight at 4 °C in a humidified chamber. The next day, sections were washed with PBS and incubated with appropriate secondary antibodies for 1 h at room temperature in the dark. Nuclei were counterstained with DAPI for 5 min, and sections were mounted with antifade mounting medium. Images were acquired using a fluorescence microscope (Olympus, Japan).

**Multiplex immunohistochemical (mIHC)**

Tissue sections were incubated at 65°C for 2 hours, followed by dewaxing in xylene for 30 minutes, then they were rehydrated through a series of graded ethanol and fixed in 10% formalin. Antigen retrieval was performed using 1× EDTA buffer in a microwave. The sections were incubated with 10% goat serum for 10 minutes to block non-specific binding sites, then incubated with the appropriately diluted primary antibody at 37°C for 30 minutes and the corresponding secondary antibody at room temperature for 10 minutes. They were then incubated with the TG TSA fluorochromes (TG520N, TG570N, TG620N, TG700N) for 10 minutes at room temperature. Antigen retrieval with sodium citrate buffer was performed between rounds of tyramide signal amplification to prevent cross-reactivity. Finally, nuclear staining was incubated with DAPI for 15 minutes. Image acquisition was performed using TissueFAXS Spectra (TG). The fluorescence intensity of each marker was quantified using Strataquest software (TG). Positive cells were detected using the 'Cell Masks' or 'Nucleus Masks' algorithm in Strataquest software, with the positive cell ratio calculated based on consistent fluorescence intensity and area thresholds. Finally, the risk score for each sample, correlation coefficients, and positive cell ratio were utilized to determine. The antibodies used are shown in Table S8.

**Terminal deoxynucleotidyl transferase-mediated dUTP Nick-End Labeling (TUNEL) assay**

Apoptotic cells were detected using a TUNEL Apoptosis Detection Kit (FITC, Biosharp, BL645, China) according to the manufacturer’s protocol. Briefly, paraffin-embedded liver sections (4 μm) were deparaffinized, rehydrated, and treated with proteinase K at 37 °C for 30 min. After PBS washing, sections were incubated with a TdT reaction mixture containing Biotin-dUTP and terminal deoxynucleotidyl transferase (TdT) at 37 °C for 1 h in the dark. Subsequently, sections were incubated with streptavidin-FITC solution for 30 min at 37 °C, followed by counterstaining with DAPI. TUNEL-positive nuclei (green fluorescence) were visualized under a fluorescence microscope (Olympus, Japan). Negative controls were prepared by omitting TdT enzyme, and DNase I–treated sections served as positive controls.

**Sirius Red Staining**

Collagen deposition was evaluated by Sirius Red staining using a Sirius Red Staining Kit (Phygene, PH1099, China) according to the manufacturer’s instructions. Briefly, paraffin-embedded liver tissue sections (4 μm) were deparaffinized, rehydrated, and incubated with Sirius Red solution for 1 h at room temperature. After rinsing with running water, nuclei were counterstained with Mayer’s hematoxylin for 8-10 min, followed by washing for 10 min. Sections were then dehydrated, cleared, and mounted with neutral resin. Collagen fibers were stained red, while other tissue components appeared yellow. Microscopic examination and image capture were conducted to document the results.

**In vivo siRNA targeting YAP and TAZ.**

To specifically inhibit YAP and TAZ *in vivo*, we employed an siRNA-based approach. Following an *in vitro* screening for efficacy, siYAP no.1 (CCAACCAGCAGCAGCAAAT) and siTAZ no. 1 (CAGAATGACTTTAGAGAAT) were selected for *in vivo* use. Approximately one week after hydrodynamic transfection of the *Mettl1* plasmid, each mouse received tail-vein injections (days 8 and 15) of 10 nmol of the respective siRNA (siYAP: cat. no. siB160328101028; siTAZ: cat. no. siB14418153031; both from RiboBio, China) in 200 μL of saline. Mice were then subjected to 70% PHx on day 28, and tissues were harvested 48 hours post-operation for analysis.

**YAP luciferase reporter construct**

The coding sequences of wild-type YAP (WT-YAP) and a synonymous mutant YAP (MUT-YAP) were cloned and inserted into the pmirGLO plasmid vector between the start codon (ATG) and the firefly luciferase (F-luc) reporter gene. The synonymous mutations introduced into the MUT-YAP coding sequence included the following codon substitutions: GUG to GUC (11 sites), CCA to CCC (11 sites), CCU to CCC (15 sites), GCA to GCC (8 sites), and CCG to CCC (12 sites). These mutations did not alter the amino acid sequence of YAP. The resulting plasmid constructs were transfected into METTL1-knockdown MIHA cells. The relative translation efficiency (TE) of WT-YAP and MUT-YAP was assessed by measuring firefly luciferase activity normalized to Renilla luciferase (R-luc) activity.

**Dual-Luciferase Reporter Assay**

Luciferase activity was measured using the Dual-Luciferase^®^ Reporter Assay System (Promega, E1980, Madison, WI, USA) according to the manufacturer’s protocol. Briefly, transfected cells were lysed in 1× Passive Lysis Buffer (PLB) for 15 min at room temperature with gentle shaking. Cell lysates (20 μl) were mixed with 100 μl of Luciferase Assay Reagent II (LAR II) to measure firefly luciferase activity, followed by the addition of 100 μl Stop & Glo^®^ Reagent to the same sample to measure Renilla luciferase activity. Luminescence was recorded using a luminometer with a 1-2 s delay and 5-10 s integration time. Relative luciferase activity was calculated as the ratio of firefly to Renilla luciferase luminescence.

**Liquid chromatography-coupled mass spectrometry for the quantitative analysis of tRNA modifications**

Liquid chromatography-coupled tandem mass spectrometry (LC-MS/MS) was performed as previously described ^[6-7]^. Briefly, total RNA was extracted from tissue samples as described above, and tRNAs were isolated from total RNA by urea-PAGE electrophoresis. Purified tRNAs were digested into dephosphorylated nucleosides through enzymatic hydrolysis, followed by deproteinization. Nucleosides were analyzed on an Agilent 6460 triple quadrupole mass spectrometer equipped with an Agilent 1260 HPLC system (Agilent, Santa Clara, CA, USA). Data acquisition and processing were carried out using Agilent Qualitative Analysis software. Multiple reaction monitoring (MRM) was used to detect each modified nucleoside, and peak areas were normalized to the amount of input tRNA. The percentage of tRNA modification was calculated as the ratio of the normalized peak area of a given modification to the sum of normalized peak areas of all detected nucleosides.

**RNA isolation, reverse transcription and qRT-PCR**

Following the instructions, RNA samples were extracted using TRIzol reagent (Invitrogen, USA). For reverse transcription, 2 μg of RNA per sample was used in a 20 μL reaction system with the PrimeScript RT Master Mix kit (Takara, Japan). After reverse transcription, the cDNA samples were diluted 1:20, and quantitative real-time PCR (qRT-PCR) was performed using the SYBR green (SYBR^®^ Premix Ex Taq^TM^ II kit Takara, Japan) on the LightCycler 480 Real-Time PCR System (Roche, USA). Each sample was repeated three times, and β-actin served as the endogenous control to normalize the expression levels of target genes across different groups. The relative mRNA expression levels of the target genes were calculated using the 2^−ΔΔCt^ method. The details of primers are shown in Table S9.

**Polysome profiling**

Polysome profiling was performed as described previously ^[4]^. *In vitro*, cells were lysed in polysome lysis buffer (50mM MOPS, 15mM MgCl2, 150mM NaCl, 100μg/mL cycloheximide, 1mg/mL heparin, 1mM benzamidine, 0.5%Triton X-100, 2mM PMSF, and 200U/mL RNase inhibitor) for 30 minutes on ice. As for liver tissue, tissues were immediately frozen in liquid nitrogen and ground to powder in liquid nitrogen with a mortar and pestle, then dissolved in 1.2ml Polysome lysis buffer, followed by cell lysis on ice for 30 minutes, then centrifuged at 13,000 g for 15 minutes at 4°C. Subsequently, a gradient sucrose solution was prepared using polysome buffer (50mM MOPS, 15mM MgCl_2_, 150mM NaCl, 100μg/mL cycloheximide), and 1ml of the cytoplasmic extract was layered onto an 11ml 10%-50% sucrose gradient. The samples were centrifuged at 36,000 rpm at 4°C for 2.5 hours in an SW41 rotor (Beckman Coulter, USA).

The separated samples were fractionated using the BR-188 Density Gradient Fractionation System (Brandel) at a rate of 0.75 ml/minute, while continuously monitoring the OD 254 values. Subsequently, ribosome profiles were generated based on the obtained OD 254 values, with single ribosome peaks and polysome peaks annotated. Mono- and polysome fractions were collected for RNA isolation to study the relative distribution of YAP and TAZ mRNA. RNA isolation and qRT-PCR were performed as described above.

**Ribosome-nascent chain-complex-bound mRNA (RNC) extraction and qRT-PCR**

The steps for the RNC extraction procedure were performed as previously described ^[8]^. Briefly, cells were treated with 100 μg/mL cycloheximide for 15min at 37°C, and incubated with 1.2ml cell lysis buffer (1% Triton X-100 in ribosome buffer (RB buffer): 15mM MgCl_2_, 20mM HEPES-KOH (pH 7.4), 200mM KCl, 100 μg/mL cycloheximide and 2mM dithiothreitol) on ice for 30 min. Cell debris was removed by centrifugation at 16,200 g for 10 min at 4°C. 80% of the extraction was transferred onto a layer of 10ml of sucrose buffer (30% sucrose in RB buffer), the remaining extraction served as the input control. Then the RNCs pellets containing the polysome fractions were collected by ultra-centrifugation at 185,000g, 5 hours at 4°C. Finally, RNA samples were isolated from the input and RNC samples and extracted using the TRIzol reagent (Invitrogen, USA). After reverse transcription, the cDNA samples were used for qRT-PCR via SYBR green (SYBR^®^ Premix Ex Taq^TM^ II kit Takara, Japan) on the LightCycler 480 Real-Time PCR System (Roche, USA).

**Puromycin intake assay in vivo**

The puromycin intake assay was performed as reported previously ^[9]^, mice were injected via tail vein with puromycin (5mg/mL/g). Then liver tissues were collected for analysis 30 minutes later. After protein extraction from the liver tissues, Western blotting was performed using an anti-puromycin antibody (Millipore) to detect the level of puromycin incorporation.

**TRAC-seq and data analysis**

RNA samples were extracted from liver tissues in wild-type and *Mettl1* cKO mice using the TRIzol reagent, and then subjected to small RNA purification using the mirVana miRNA Isolation Kit (AM1561, Thermo Fisher Scientific). The isolated small RNAs were demethylated by using recombinant wild-type and D135S AlkB proteins to remove the majority of methylations. Then, 50% of the RNA sample was subjected to 0.1mol/L NaBH_4_ and aniline-acetate solution (H_2_O: glacial acid: aniline =7:3:1) treatment to induce reduction and cleavage at m^7^G-modified sites, and the remaining 50% of the AlkB-demethylated RNA sample served as an input sample. After purification with the Oligo Clean & Concentrator Kit, the input group and RNA samples treated with NaBH_4_ and aniline were used for constructing high-throughput sequencing libraries using the NEBNext Multiplex Small RNA Library Prep Set for Illumina Kit (New England Biolabs, USA), followed by high-throughput sequencing ^[10]^.

The analysis of tRNA expression and m^7^G modification was performed as previously described ^[4, 10-12]^. In brief, clean reads were mapped to the mouse mature tRNA sequences (<http://gtrnadb.ucsc.edu/genomes/eukaryota/Mmusc10>) ^[13]^. TRAC-seq introduces specific cleavage at m^7^G-modified sites with NaBH_4_/aniline treatment, and then adaptors were ligated to treated RNAs for library construction. The NaBH_4_/aniline treatment induced cleavage at m^7^G sites, which results in sequencing reads that start after the m^7^G sites. Then Bedtools (https://bedtools.readthedocs.io/en/latest) was used to calculate the read depth of each site (tRNA abundance) and the number of reads starting at that position (cleaved reads). The introns were removed for mature tRNA sequences, and ‘CCA’ was added to the 3’ end of the tRNA gene sequences. The clean sequencing data were then mapped to the tRNA sequences by Bowtie2 (<http://bowtie-bio.sourceforge.net>). The read counts of tRNA are then normalized as RPKM by the total number of small RNA reads that are matched to tRNAs in each sample.

The proportion of cleaved sequences for each tRNA in the control and reduced cleavage groups was calculated, defining this ratio as the cleavage score using the following formula:

*Cleavage score_i_*​= $\frac{log2 ({Cleavage ratio}_{treat})}{log2 ({Cleavage ratio}_{input})}$

The cleavage score, which indicates the m^7^G modification level, was defined as the ratio of cleaved reads in the treated samples to the read depth in the input samples. The positions 42-49, each with a cleavage score >3, were considered candidate m^7^G sites.

**Ribosome sequencing (Ribo-seq) and data analysis**

The Ribo-seq was performed as previously described ^[14]^. Liver tissues were immediately frozen in liquid nitrogen and ground to powder in liquid nitrogen with a mortar and pestle, then dissolved in 400µL of lysis buffer. Resuspended extracts in lysis buffer were transferred to new microtubes, pipetted several times, and incubated on ice for 10 minutes. The cells were then triturated ten times through a 26-G needle. The lysate was centrifuged at 20,000 g for 10 minutes at 4°C, and the supernatant was collected. To prepare ribosome footprints (RFs), 10 µL of RNase I (NEB, Ipswich, MA, USA) and 6 µL of DNase I (NEB, Ipswich, MA, USA) were added to 400 µL of lysate, which was then incubated for 45 minutes at room temperature with gentle mixing on a Nutator mixer. Nuclease digestion was stopped by adding 10 µL of SUPERase·In RNase inhibitor (Ambion, Austin, TX, USA). Size exclusion columns (Illustra MicroSpin S-400 HR Columns; GE Healthcare; catalog no. 27-5140-01) were equilibrated with 3 mL of polysome buffer by gravity flow and centrifuged at 600 g for 4 minutes at room temperature. 100 μL of digested RFs were added to the column and centrifuged at 600 g for 2 minutes. Next, 10 μL of 10% (wt/vol) SDS was added to the elution, and RFs larger than 17 nt were isolated using the RNA Clean and Concentrator-25 kit (Zymo Research; R1017). rRNA was removed following a previously reported method ^[15]^. Briefly, short (50-80 bases) antisense DNA probes complementary to rRNA sequences were added to the RF solution, followed by RNase H (NEB, Ipswich, MA, USA) and DNase I (NEB, Ipswich, MA, USA) digestion to remove rRNA and residual DNA probes. The RFs were further purified using magnetic beads (Vazyme, Nanjing, Jiangsu, China). Following ribosome footprint isolation, Ribo-seq libraries were constructed using the NEBNext^®^ Multiple Small RNA Library Prep Set for Illumina^®^ (catalog no. E7300S, E7300L). Adapters were added to both ends of the RFs, followed by reverse transcription and PCR amplification. The 140-160 bp size PCR products were enriched to generate a cDNA library and sequenced using the Illumina HiSeqTM X10 platform by Gene Denovo Biotechnology Co. (Guangzhou, China).

**Kyoto Encyclopedia of Genes and Genomes (KEGG) pathway analysis and Gene Set Enrichment Analysis (GSEA)**

Gene Set Enrichment Analysis (GSEA) ^[16]^ was performed using the KEGG (Kyoto Encyclopedia of Genes and Genomes) database (https://www.kegg.jp/kegg/pathway.html) with the clusterProfiler package ^[17]^ in R between mice before and after hepatectomy. Briefly, all the detected genes by proteomics ranked according to their log2 fold change between mice before and after hepatectomy were used as input for GSEA.

GSEA using the KEGG database and YAP/TAZ signature gene set ^[18]^ was also applied to identify whether a set of genes in specific pathways showed significant TE differences in the regenerating liver tissues of control and *Mettl1* cKO mice. Identified by ribo-seq, TE-down mRNAs ranked according to their TE fold change between the regenerating liver tissues of control and *Mettl1* cKO mice were used as input for GSEA. Pathways with a p-value < 0.05 were classified as significantly enriched.

**Cell lines and culture**

The immortalized human liver cell line MIHA was kindly provided by Dr. J.R. Chowdhury, Albert Einstein College of Medicine (New York, USA). Cells were cultured in RPMI-1640 (Procell, China) with 10% FBS (Gibco, USA) and 1% penicillin-streptomycin (Gibco, USA) and cultured in a 5% CO_2_ cell culture incubator (Thermo Scientific, USA) at 37 °C.

**Lentiviral transduction, shRNA/sgRNA knockdown and overexpression**

Lentiviral vectors expressing pLKO.1 negative control sh/sgRNA (NC), sh/sgRNA, overexpression (WT), and inactive mutant constructs (Mut) targeting METTL1 were obtained from IGE Biotechnology Co.Ltd. Using Lipofectamine 3000 (Invitrogen, Waltham, MA, USA), co-transfect the RNA construct along with the packaging plasmid (pCMV-ΔR8.9) and the envelope plasmid (pCMV-VSVG) into HEK 293T cells. After incubating for 48 hours, Viruses were collected by centrifuging at 2,000 rpm for 5 minutes at room temperature and infecting MIHA cells with 8-10 μL of Polybrene. After 48 hours of virus transduction, a medium containing puromycin (5 μg/mL) was used to select METTL1 knockdown cells or METTL1 overexpression cells for 48 hours. Regularly observe the cell status, eventually obtaining stable METTL1 knockdown and overexpression cell lines. The sequences of RNA primers are shown in Table S5. For the overexpression of proAGG, three repeats of proAGG gene sequence were synthesized and cloned into the SpeI and ApaI sites of pmirGLO. Then the pmirGLO-3x proAGG vector and control vector were transfected into cells using Lipofectamine 3000 following the manufacturer’s instructions.

**References**

[1] N. N. Rahbari, O. J. Garden, R. Padbury, et al., "*Posthepatectomy liver failure: a definition and grading by the International Study Group of Liver Surgery (ISGLS)"*, *Surgery* **2011**, *149* (5), 713-724, <https://doi.org/10.1016/j.surg.2010.10.001>.

[2] X. Chen, D. F. Calvisi, "*Hydrodynamic transfection for generation of novel mouse models for liver cancer research"*, *Am J Pathol* **2014**, *184* (4), 912-923, <https://doi.org/10.1016/j.ajpath.2013.12.002>.

[3] S. W. Kim, Z. Li, P. S. Moore, et al., "*A sensitive non-radioactive northern blot method to detect small RNAs"*, *Nucleic Acids Res* **2010**, *38* (7), e98, <https://doi.org/10.1093/nar/gkp1235>.

[4] S. Lin, Q. Liu, V. S. Lelyveld, et al., "*Mettl1/Wdr4-Mediated m(7)G tRNA Methylome Is Required for Normal mRNA Translation and Embryonic Stem Cell Self-Renewal and Differentiation"*, *Mol Cell* **2018**, *71* (2), 244-255 e245, <https://doi.org/10.1016/j.molcel.2018.06.001>.

[5] R. Kolde, S. Laur, P. Adler, J. Vilo, "*Robust rank aggregation for gene list integration and meta-analysis"*, *Bioinformatics* **2012**, *28* (4), 573-580, <https://doi.org/10.1093/bioinformatics/btr709>.

[6] D. Su, C. T. Chan, C. Gu, et al., "*Quantitative analysis of ribonucleoside modifications in tRNA by HPLC-coupled mass spectrometry"*, *Nat Protoc* **2014**, *9* (4), 828-841, <https://doi.org/10.1038/nprot.2014.047>.

[7] M. Yan, Y. Wang, Y. Hu, et al., "*A high-throughput quantitative approach reveals more small RNA modifications in mouse liver and their correlation with diabetes"*, *Anal Chem* **2013**, *85* (24), 12173-12181, <https://doi.org/10.1021/ac4036026>.

[8] T. Wang, Y. Cui, J. Jin, et al., "*Translating mRNAs strongly correlate to proteins in a multivariate manner and their translation ratios are phenotype specific"*, *Nucleic Acids Res* **2013**, *41* (9), 4743-4754, <https://doi.org/10.1093/nar/gkt178>.

[9] E. K. Schmidt, G. Clavarino, M. Ceppi, P. Pierre, "*SUnSET, a nonradioactive method to monitor protein synthesis"*, *Nat Methods* **2009**, *6* (4), 275-277, <https://doi.org/10.1038/nmeth.1314>.

[10] S. Lin, Q. Liu, Y. Z. Jiang, R. I. Gregory, "*Nucleotide resolution profiling of m(7)G tRNA modification by TRAC-Seq"*, *Nature protocols* **2019**, *14* (11), 3220-3242, <https://doi.org/10.1038/s41596-019-0226-7>.

[11] A. E. Cozen, E. Quartley, A. D. Holmes, et al., "*ARM-seq: AlkB-facilitated RNA methylation sequencing reveals a complex landscape of modified tRNA fragments"*, *Nat Methods* **2015**, *12* (9), 879-884, <https://doi.org/10.1038/nmeth.3508>.

[12] G. Zheng, Y. Qin, W. C. Clark, et al., "*Efficient and quantitative high-throughput tRNA sequencing"*, *Nat Methods* **2015**, *12* (9), 835-837, <https://doi.org/10.1038/nmeth.3478>.

[13] T. M. Lowe, S. R. Eddy, "*tRNAscan-SE: a program for improved detection of transfer RNA genes in genomic sequence"*, *Nucleic Acids Res* **1997**, *25* (5), 955-964, <https://doi.org/10.1093/nar/25.5.955>.

[14] N. T. Ingolia, G. A. Brar, S. Rouskin, A. M. McGeachy, J. S. Weissman, "*The ribosome profiling strategy for monitoring translation in vivo by deep sequencing of ribosome-protected mRNA fragments"*, *Nat Protoc* **2012**, *7* (8), 1534-1550, <https://doi.org/10.1038/nprot.2012.086>.

[15] J. D. Morlan, K. Qu, D. V. Sinicropi, "*Selective depletion of rRNA enables whole transcriptome profiling of archival fixed tissue"*, *PLoS One* **2012**, *7* (8), e42882, <https://doi.org/10.1371/journal.pone.0042882>.

[16] A. Subramanian, P. Tamayo, V. K. Mootha, et al., "*Gene set enrichment analysis: a knowledge-based approach for interpreting genome-wide expression profiles"*, *Proc Natl Acad Sci U S A* **2005**, *102* (43), 15545-15550, <https://doi.org/10.1073/pnas.0506580102>.

[17] T. Wu, E. Hu, S. Xu, et al., "*clusterProfiler 4.0: A universal enrichment tool for interpreting omics data"*, *Innovation (Camb)* **2021**, *2* (3), 100141, <https://doi.org/10.1016/j.xinn.2021.100141>.

[18] J. Dong, G. Feldmann, J. Huang, et al., "*Elucidation of a universal size-control mechanism in Drosophila and mammals"*, *Cell* **2007**, *130* (6), 1120-1133, <https://doi.org/10.1016/j.cell.2007.07.019>.

**Supplementary Figure Legends**

**Figure S1. Significant Proliferation of Residual Liver after 70% PHx**

**A-B.** Representative images show liver regeneration (A) and quantitative analysis of the liver-to-body weight ratio (LW/BW%) (B) in mice at different time points (D0-D14) after PHx.

**Figure S2. Biochemical and histological evaluation of liver injury and regeneration in *Mettl1* cKO mice after PHx**

**A**. Serum levels of ALT/AST/TBIL/ALB in *Mettl1* conditional knockout (cKO) mice.

**B-C**. Representative images (B) and quantification (C) of Sirius Red staining.

**D-E**. Representative images (D) and quantification (E) of TUNEL staining.

**F-G**. Representative images (F) and quantitative analysis (G) of mIHC staining for METTL1, CK19, α-SMA in paraffin sections of the liver at day 2 after PHx.

Scale bars for IF and mIHC: 50 μm. Data are presented as mean ± SEM. Significance is indicated as follows: **P*<0.05, ***P*<0.01, ****P*<0.001, ns indicates no significant difference.

**Figure S3. Liver function parameters in control mice with or without AAV8 injection**

**A.** Serum levels of ALT/AST/TBIL/ALB in control mice with or without AAV8 injection.

Significance is indicated as follows: **P*<0.05, ***P*<0.01, ****P*<0.001, ns indicates no significant difference.

**Figure S4. METTL1 Knockdown Impairs Liver Regeneration After PHx**

**A.** Experimental schematic: 4-5 weeks-old C57BL/6 mice were injected via tail vein with AAV8-shNC or AAV8-shM1, and 70% PHx was performed 4 weeks later, followed by tissue collection 48 hours after PHx.

**B.** Quantification of AAV8 accumulation in the liver using In Vivo Living Image software 4.5.5 (PerkinElmer, Waltham, Mass), with data presented as mean radiance (photons/sec/cm²/steradian).

**C.** Western blot analysis confirming the knockdown of METTL1 protein levels in liver tissues.

**D.** Ratio of LW to BW in the above groups at D2 after PHx (n=5).

**E.** Western blot analysis of CyclinB1, CyclinD1, PCNA, and METTL1 in liver tissues at D2 after PHx.

**F-G.** IHC staining (F) and quantitative analysis (G) of METTL1, PCNA, Ki67, and BrdU in liver tissues at D2 after PHx.

β-Actin was used as a loading control for Western blot analysis. Scale bars for IHC: 50 μm. Data are presented as mean ± SEM. Significance is indicated as follows: **P*<0.05, ***P*<0.01, ****P*<0.001, ns indicates no significant difference.

**Figure S5. METTL1 regulates YAP/TAZ translation through Hippo pathway and m^7^G modification**

**A.** Western blot analysis of Hippo pathway genes in liver tissue after PHx.

**B.** Correlation analysis between the Ribo-seq and proteome data of genes in YAP/TAZ pathways.

**C.** Western blot analysis of YAP and TAZ protein expression in METTL1-knockdown MIHA cells, rescue with wild-type (m^7^G) or m^7^G-deficient mutant (m^7^C) ProAGG.

**D.** A dual-luciferase reporter assay was performed to compare the translation of wild-type YAP and a synonymous mutant sequence of YAP. Translational ratio was determined by normalizing the firefly luciferase (F-luc) activity (which reports YAP translation) to the Renilla luciferase (R-luc) activity (used as an internal control).

β-Actin and U6 were used as a loading control for Western blot and Northern blot analysis. Data are presented as mean ± SEM. Significance is indicated as follows: **P*<0.05, ***P*<0.01, ****P*<0.001, ns indicates no significant difference.

**Figure S6. YAP/TAZ mediates the regulatory effects of METTL1 on liver regeneration after PHx**.

**A.** Experimental schematic: 4-5 weeks-old C57BL/6 mice were injected with Mettl1 plasmid (oeM1) or empty plasmid (oeNC) via hydrodynamic injection, each mouse received two injections of 10 nmol siYAP/siTAZ or negative control siRNA in 200 μL of saline via the tail vein on days 8 and 15. Then, two weeks later (on day 28), partial hepatectomy was performed in these mice, followed by tissue collection 48 hours after PHx.

**B-C.** Ratio of LW to BW **(B)** and protein expression level of proliferation-related markers **(C)** in siRNA-mediated knockdown mice models targeting YAP and TAZ individually and together.

**D-E.** Relative YAP mRNA expression levels and serum levels of ALT/AST/TBIL/ALB in mouse model established by hydrodynamic injection of Yap.

**F-G.** Relative YAP mRNA expression levels and serum levels of ALT/AST/TBIL/ALB in mouse livers following AAV8-mediated Yap delivery.

β-Actin was used as a loading control for Western blot analysis. Data are presented as mean ± SEM. Significance is indicated as follows: **P*<0.05, ***P*<0.01, ****P*<0.001, ns indicates no significant difference.

**Figure S7. Relative mRNA Expression Levels of YAP Downstream Proliferation-Related Target Genes After PHx in Different Mouse Models**

**A-D.** qRT-PCR analysis of relative mRNA expression levels of *Areg, Birc5, Ccnb1, Ctgf, Cyr61*, and *Foxm1* in liver tissues of various mouse models after PHx. **A:** Ctrl model; **B:** Ctrl and cKO model; **C:** Mice with METTL1 knockdown via AAV8; **D:** Mice with METTL1 overexpression.

Data are presented as mean ± SEM. Significance is indicated as follows: **P*<0.05, ***P*<0.01, ****P*<0.001, ns indicates no significant difference.

**Figure S8. Graphical Summary:** **The model for METTL1-Mediated tRNA m^7^G Modification in Regulation of Liver Regeneration after PHx.**

The levels of METTL1 and tRNA m^7^G modification were significantly increased after hepatectomy. METTL1, along with its mediated tRNA m^7^G modification, selectively regulates the translation efficiency of YAP/TAZ, the key proteins of the Hippo pathway, through a codon frequency-dependent mechanism, and promotes the transcription of downstream proliferation and cycle-related target genes, thereby promoting the regeneration of remnant liver.

**Figure S9. YAP expression in different mouse models.**

Relative protein expression levels and mRNA expression levels of YAP in various mouse models after PHx. **A**-B: WT mice model; **C-D:** Ctrl and cKO model; **E-F:** Mice with METTL1 overexpression.

Data are presented as mean ± SEM. Significance is indicated as follows: **P*<0.05, ***P*<0.01, ****P*<0.001, ns indicates no significant difference.
