# Supplementary material for: METTL1‐Mediated M7G tRNA Modification Promotes Residual Liver Regeneration After Hepatectomy via Translational Control
Source: Adv Sci (Weinh). 2025 Dec 8;13(12):e07329. doi: 10.1002/advs.202507329 (PMC12948282; doi:10.1002/advs.202507329)
Supplement: Supplementary file 2 — Supporting Information [file ADVS-13-e07329-s004.docx]

**Supplemental Tables**

**Table S1. Univariate linear regression analysis of clinical variables associated with liver regeneration index**

| Variables | Estimate | 2.5% CI | 97.5% CI | *p* value |
| --- | --- | --- | --- | --- |
| Sex: male | 0.0496 | -0.0226 | 0.1219 | 0.17 |
| Age≥60y | -0.0261 | -0.0944 | 0.0421 | 0.45 |
| BMI | -0.0012 | -0.0109 | 0.0085 | 0.80 |
| Laparoscope | 0.0116 | -0.0502 | 0.0736 | 0.71 |
| Blood loss (mL) | 5.9225 | -5.8818E-05 | 0.0002 | 0.32 |
| Liver cirrhosis | -0.0052 | -0.0829 | 0.0725 | 0.89 |
| CSPH | 0.0032 | -0.0802 | 0.0868 | 0.94 |
| HBsAg | 0.0235 | -0.0424 | 0.0896 | 0.48 |
| ALT (U/L) | -8.2842E-05 | -0.0005 | 0.0004 | 0.72 |
| AST (U/L) | -0.0003 | -0.0009 | 0.0002 | 0.21 |
| GGT (U/L) | -3.5361E-05 | -0.0002 | 0.0001 | 0.67 |
| LDH (U/L) | -2.0658E-05 | -4.5055E-05 | 3.7383 | 0.10 |
| ALP (U/L) | -0.0002 | -0.0003 | 3.0392E-05 | 0.10 |
| CHE (U/L) | 1.2545E-06 | -1.6444E-05 | 1.8953E-05 | 0.89 |
| TP (g/L) | 0.0018 | -0.0032 | 0.0068 | 0.47 |
| ALB (g/L) | 0.0038 | -0.0034 | 0.0110 | 0.29 |
| PA (mg/L) | 0.0003 | -0.0001 | 0.0008 | 0.15 |
| TBIL (μmol/L) | -0.0003 | -0.0008 | 0.0002 | 0.31 |
| PLT (*10^9/L) | -0.0001 | -0.0004 | 0.0002 | 0.45 |
| AFP≥400 | -0.0161 | -0.0813 | 0.0491 | 0.62 |
| ANRI | -0.0016 | -0.0044 | 0.0013 | 0.28 |
| ALRI | -0.0006 | -0.0014 | 0.0003 | 0.17 |
| Major complications | -0.0386 | -0.1133 | 0.0361 | 0.31 |

BMI: Body Mass Index; CSPH: Clinically Significant Portal Hypertension; ALT: alanine transaminase; AST: aspartate transaminase; GGT: Gamma-Glutamyl Transferase; LDH: Lactate Dehydrogenase; ALP: Alkaline Phosphatase; CHE: Cholinesterase; TP: Total protein; ALB: Albumin; PA: Prealbumin; TBIL: Total bilirubin; PLT: Platelet count; AFP: Alpha-fetoprotein; ANRI: Aspartate aminotransferase to the neutrophil ratio; ALRI: aspartate aminotransferase to the lymphocyte ratio.

**Table S2. Univariate and multivariate linear regression analysis of clinical variables associated with the METTL1 level**

|  | Univariate |  | multivariate |  |
| --- | --- | --- | --- | --- |
| Variables | Estimate | *p* value | Estimate | *p* value |
| Sex: male | -0.0020 | 0.41 |  |  |
| Age≥60y | -0.0017 | 0.45 |  |  |
| BMI | -6.12E-05 | 0.85 |  |  |
| Laparoscope | -0.0006 | 0.77 |  |  |
| Blood loss (mL) | 2.0037E-06 | 0.61 |  |  |
| Liver cirrhosis | -0.0028 | 0.26 |  |  |
| CSPH | -0.0015 | 0.59 |  |  |
| HBsAg | -0.0010 | 0.63 |  |  |
| ALT (U/L) | 6.7231E-06 | 0.66 |  |  |
| AST (U/L) | 2.3667E-05 | 0.18 |  |  |
| GGT (U/L) | 3.8790E-07 | 0.94 |  |  |
| LDH (U/L) | -7.91E-07 | 0.34 |  |  |
| ALP (U/L) | 4.1397E-06 | 0.50 |  |  |
| CHE (U/L) | -1.61E-07 | 0.78 |  |  |
| TP (g/L) | -6.08E-05 | 0.71 |  |  |
| ALB (g/L) | -0.0002 | 0.31 |  |  |
| PA (mg/L) | -1.87E-05 | 0.23 |  |  |
| TBIL (μmol/L) | 3.4556E-05 | 0.04 |  |  |
| PLT (*10^9/L) | 1.3615E-05 | 0.16 |  |  |
| AFP≥400 | -0.0010 | 0.64 |  |  |
| ANRI | -7.59E-06 | 0.94 |  |  |
| ALRI | 4.5296E-05 | 0.10 |  |  |
| Major complications | -0.0013 | 0.60 |  |  |

BMI: Body Mass Index; CSPH: Clinically Significant Portal Hypertension; ALT: alanine transaminase; AST: aspartate transaminase; GGT: Gamma-Glutamyl Transferase; LDH: Lactate Dehydrogenase; ALP: Alkaline Phosphatase; CHE: Cholinesterase; TP: Total protein; ALB: Albumin; PA: Prealbumin; TBIL: Total bilirubin; PLT: Platelet count; AFP: Alpha-fetoprotein; ANRI: Aspartate aminotransferase to the neutrophil ratio; ALRI: aspartate aminotransferase to the lymphocyte ratio.

**Table S3. Univariate and multivariate linear regression analysis of clinical variables associated with YAP level**

|  | Univariate |  | multivariate |  |
| --- | --- | --- | --- | --- |
| Variables | Estimate | *p* value | Estimate | *p* value |
| Sex: male | -0.0004 | 0.99 |  |  |
| Age≥60y | -0.0025 | 0.95 |  |  |
| BMI | 0.0027 | 0.60 |  |  |
| Laparoscope | -0.0470 | 0.16 |  |  |
| Blood loss (mL) | -5.74E-05 | 0.37 |  |  |
| Liver cirrhosis | -0.0184 | 0.66 |  |  |
| CSPH | -0.0290 | 0.52 |  |  |
| HBsAg | -0.0093 | 0.80 |  |  |
| ALT (U/L) | 9.67068E-05 | 0.70 |  |  |
| AST (U/L) | 5.0996E-05 | 0.86 |  |  |
| GGT (U/L) | 7.9711E-05 | 0.36 |  |  |
| LDH (U/L) | -4.48E-06 | 0.74 |  |  |
| ALP (U/L) | 3.7672E-05 | 0.71 |  |  |
| CHE (U/L) | -2.22E-05 | 0.08 |  |  |
| TP (g/L) | -0.0017 | 0.51 |  |  |
| ALB (g/L) | -0.01012 | 0.01 | -6.602e-03 | 0.10 |
| PA (mg/L) | -0.00056 | 0.03 | 4.918e-05 | 0.86 |
| TBIL (μmol/L) | 9.98177E-05 | 0.71 |  |  |
| PLT (*10^9/L) | 0.0005 | <0.001 | 2.547e-04 | 0.19 |
| AFP≥400 | 0.0070 | 0.84 |  |  |
| ANRI | -0.0005 | 0.72 |  |  |
| ALRI | 4.0302E-05 | 0.93 |  |  |
| Major complications | 0.0159 | 0.70 |  |  |

BMI: Body Mass Index; CSPH: Clinically Significant Portal Hypertension; ALT: alanine transaminase; AST: aspartate transaminase; GGT: Gamma-Glutamyl Transferase; LDH: Lactate Dehydrogenase; ALP: Alkaline Phosphatase; CHE: Cholinesterase; TP: Total protein; ALB: Albumin; PA: Prealbumin; TBIL: Total bilirubin; PLT: Platelet count; AFP: Alpha-fetoprotein; ANRI: Aspartate aminotransferase to the neutrophil ratio; ALRI: aspartate aminotransferase to the lymphocyte ratio.

**Table S4. Characteristics of patients**

| Variables | N=65 | Non-PHLF | PHLF | *P* value |
| --- | --- | --- | --- | --- |
|  |  | n=50 | n=15 |  |
| Sex: male, n (%) | 51(78.5) | 38(76.0) | 13(86.7) | 0.491 |
| Age≥60y, n (%) | 17(26.2) | 14(28.0) | 3(20.0) | 0.741 |
| BMI | 22.47[20.37,23.94] | 22.46[20.40,23.66] | 22.84[19.70,25.43] | 0.779 |
| TLV (mL) | 1465.28[1254.82,1647.36] | 1466.92[1195.29,1675.78] | 1460.12[1323.87,1579.38] | 0.652 |
| eRLV (mL) | 720.20[552.60,982.70] | 739.54[541.92,954.72] | 669.65[569.40,947.76] | 0.755 |
| FLV (mL) | 627.89[486.77,820.88] | 624.25[493.49,836.89] | 698.65[480.75,800.38] | 0.901 |
| FLV 1month (mL) | 950.53[785.45,1147.64] | 1004.23[797.61,1190.24] | 853.92[688.27,960.88] | 0.081 |
| RegenLV (mL) | 254.79[186.34,414.99] | 298.92[204.90,438.83] | 194.86[136.28,219.52] | 0.003 |
| RegenIndex | 0.19[0.13,0.29] | 0.21[0.15,0.30] | 0.12[0.10,0.22] | 0.015 |
| Laparoscope, n (%) | 25(38.5) | 18(36.0) | 7(46.7) | 0.549 |
| Tumor size (cm) | 6.40[4.00,9.70] | 6.35[4.28,9.67] | 8.30[3.80,9.98] | 0.846 |
| Tumor number, n (%) |  |  |  | 0.044 |
| 1 | 43(66.2) | 37(74.0) | 6(40.0) |  |
| 2 | 10(15.4) | 5(10.0) | 5(33.3) |  |
| 3 | 2(3.1) | 1(2.0) | 1(6.7) |  |
| ≥4 | 10(15.4) | 7(14.0) | 3(20.0) |  |
| Blood loss (mL) | 200.00[100.00,500.00] | 200.00[100.00,487.50] | 300.00[125.00,600.00] | 0.473 |
| Liver cirrhosis, n (%) | 12(18.5) | 7(14.0) | 5(33.3) | 0.128 |
| CSPH, n (%) | 10(15.4) | 7(14.0) | 3(20.0) | 0.685 |
| HBsAg, n (%) | 46(70.8) | 33(66.0) | 13(86.7) | 0.196 |
| ALT (U/L) | 27.00[20.00,44.00] | 26.50[19.25,42.25] | 31.00[24.50,44.50] | 0.409 |
| AST (U/L) | 32.00[26.00,45.00] | 30.50[25.25,42.25] | 43.00[33.00,53.00] | 0.074 |
| GGT (U/L) | 94.00[48.00,164.00] | 97.50[47.25,196.25] | 77.00[54.00,131.00] | 0.575 |
| LDH (U/L) | 200.00[184.00,245.00] | 202.00[185.25,237.75] | 200.00[170.00,286.50] | 0.821 |
| ALP (U/L) | 105.00[84.00,156.00] | 104.00[78.75,155.50] | 105.00[88.50,154.00] | 0.726 |
| CHE (U/L) | 6262.00[5090.00,7486.00] | 6428.50[5241.00,7872.00] | 6129.00[5050.50,6476.50] | 0.127 |
| TP (g/L) | 67.80[63.30,71.80] | 68.20[63.23,71.00] | 67.10[64.05,72.45] | 0.932 |
| ALB (g/L) | 38.60[35.30,40.30] | 38.35[35.30,40.25] | 39.10[34.70,40.25] | 0.994 |
| PA (mg/L) | 190.00[152.00,233.00] | 192.50[158.00,239.00] | 161.00[143.50,220.50] | 0.252 |
| TBIL (μmol/L) | 14.80[11.00,18.50] | 14.45[10.55,18.65] | 16.30[11.65,17.30] | 0.827 |
| Hb (g/L) | 136.00[113.00,150.00] | 136.00[113.00,150.50] | 143.00[112.00,149.50] | 0.889 |
| PLT (*10^9/L) | 212.00[165.00,283.00] | 218.00[184.50,283.75] | 200.00[139.50,241.00] | 0.118 |
| PT (s) | 12.00[11.40,12.60] | 11.70[11.30,12.47] | 12.50[12.25,12.70] | 0.029 |
| Fbg (g/L) | 3.12[2.67,4.50] | 3.19[2.76,4.39] | 2.97[2.64,4.60] | 0.926 |
| AFP ≥400, n (%) | 20(30.8) | 13(26.0) | 7(46.7) | 0.201 |
| BCLC stage, n (%) |  |  |  | 0.554 |
| Stage 0/A | 28(43.1) | 23(46.0) | 5(33.3) |  |
| Stage B/C | 37(56.9) | 27(54.0) | 10(66.7) |  |
| Child-Pugh score | 5.00[5.00,6.00] | 5.00[5.00,6.00] | 5.00[5.00,5.50] | 0.59 |
| Child-Pugh grade, n (%) |  |  |  | 0.566 |
| Grade A | 61(93.8) | 46(92.0) | 15(100.0) |  |
| Grade B | 4(6.2) | 4(8.0) | 0(0.0) |  |
| ANRI | 8.83[5.99,14.15] | 7.96[5.33,11.76] | 12.28[9.87,18.87] | 0.013 |
| ALRI | 19.20[14.11,33.35] | 18.60[13.11,32.76] | 24.40[17.41,33.21] | 0.269 |

BMI: Body Mass Index; TLV: Total Liver Volume; eRLV: Estimated Residual Liver Volume; FLV 1month: Future Liver Volume 1 month after hepatectomy; RegenLV: Regenerative Liver Volume; RegenIndex: Regeneration Index; CSPH: Clinically Significant Portal Hypertension; ALT: alanine transaminase; AST: aspartate transaminase; GGT: Gamma-Glutamyl Transferase; LDH: Lactate Dehydrogenase; ALP: Alkaline Phosphatase; CHE: Cholinesterase; TP: Total protein; ALB: Albumin; PA: Prealbumin; TBIL: Total bilirubin; PLT: Platelet count; PT: Prothrombin time; Fbg: Fibrinogen; AFP: Alpha-fetoprotein; BCLC: Barcelona Clinical Liver Cancer; ANRI: Aspartate aminotransferase to the neutrophil ratio; ALRI: aspartate aminotransferase to the lymphocyte ratio; PHLF: post-hepatectomy liver failure.

**Table S5. Oligonucleotide sequences in this study**

| **The sequences of AAV8-m-Mettl1-shRNA** | |
| --- | --- |
| **No.** | Sequences (5'-3') |
| AAV8-m-Mettl1-shRNA-EGFP | GCCTCTTGAAGAGCTAAGT |
| AAV8-m-Mettl1-shNC-EGFP | TTCTCCGAACGTGTCACGTAA |
| **The sequences of AAV8-m-Mettl1-mut** | |
| **No.** | Sequences (5'-3') |
| AAV8-TBG-m-Mettl1-mut-ZsGreen-1F | CGAAGAGGATCTATTTCCGGTGAATTCGCCACCATGATGCTTGAGTTTAC |
| AAV8-TBG-m-Mettl1-mut-ZsGreen-1R | GTGTGGGGCCGGGAACGCGAAGAACATCTTTGCCAGCTGGCCCTTGCG |
| AAV8-TBG-m-Mettl1-mut-ZsGreen-2F | GTTCTTCGCGTTCCCGGCCCCACACTTTAAGCGAACGAAGCATAAATG |
| AAV8-TBG-m-Mettl1-mut-ZsGreen-2R | GTCATCGTCATCCTTGTAGTCGGATCCAGGCAGGGTGGGGTTGGGGGTC |
| **The sequences of AAV8-TBG-m-Mettl1/Yap** | |
| **No.** | Sequences (5'-3') |
| AAV8-TBG-m-Mettl1-ZsGreen-F | GAAGAGGATCTATTTCCGGTGAATTCGCCACCATGATGCTTGAGTTTAC |
| AAV8-TBG-m-Mettl1-ZsGreen-R | ATCGTCATCCTTGTAGTCGGATCCAGGCAGGGTGGGGTTGGGGGT |
| AAV8-TBG-m-Yap-ZsGreen-F | GAAGAGGATCTATTTCCGGTGAATTCGCCACCATGGAGCCCGCGCAAC |
| AAV8-TBG-m-Yap-ZsGreen-R | TCATCGTCATCCTTGTAGTCGGATCCTAACCACGTGAGAAAGCTTTC |
| **The sequences of shRNA** | |
| **No.** | Sequences (5'-3') |
| METTL1 shRNA #1 | TTTGTCCGCTTGAAATGTGGG |
| METTL1 shRNA #2 | AAATGAGTGCACATCCAGTCG |
| **The sequences of sgRNA** | |
| **No.** | Sequences (5'-3') |
| METTL1 sgNC | GTATTACTGATATTGGTGGG |
| METTL1 sgRNA | GGTTGGAGTGAGCACGTTGC |
| **The sequences of siRNA** | |
| **No.** | Sequences (5'-3') |
| YAP siRNA | CCAACCAGCAGCAGCAAAT |
| TAZ siRNA | CAGAATGACTTTAGAGAAT |
| **The sequences of METTL1-WT/Mut RNA** | |
| **No.** | Sequences (5'-3') |
| METTL1 WT | GAATTCGCCACCATGGCAGCCGAGACTCGGAACGTGGCCGGAGCAGAGGCCCCACCGCCCCAGAAGCGCTACTACCGGCAACGTGCTCACTCCAACCCCATGGCGGACCACACGCTGCGCTACCCTGTGAAGCCAGAGGAGATGGACTGGTCTGAGCTATACCCAGAGTTCTTCGCTCCACTCACTCAAAATCAGAGCCACGATGACCCAAAGGATAAGAAAGAAAAGAGAGCTCAGGCCCAAGTGGAGTTTGCAGACATAGGCTGTGGCTATGGTGGCCTGTTAGTGGAACTGTCACCGCTGTTCCCAGACACACTTATTCTGGGTCTGGAGATCCGGGTGAAGGTCTCAGACTATGTACAAGACCGGATTCGGGCCCTACGCGCAGCTCCTGCAGGTGGCTTCCAGAACATCGCCTGTCTCCGTAGCAATGCCATGAAGCACCTTCCTAACTTCTTCTACAAGGGCCAGCTGACAAAGATGTTCTTCCTCTTCCCCGACCCACATTTCAAGCGGACAAAGCACAAGTGGCGAATCATCAGTCCCACCCTGCTAGCAGAATATGCCTACGTGCTAAGAGTTGGGGGGCTGGTGTATACCATAACCGATGTGCTGGAGCTACACGACTGGATGTGCACTCATTTCGAAGAGCACCCACTGTTTGAGCGTGTGCCTCTGGAGGACCTGAGTGAAGACCCCGTTGTGGGACATCTAGGCACCTCAACTGAGGAGGGGAAGAAAGTTCTACGTAATGGAGGGAAGAATTTCCCAGCCATCTTCCGAAGAATACAAGATCCCGTCCTCCAGGCAGTGACCTCCCAAACCAGCCTGCCTGGTCACTGAGGATCC |
| METTL1 Mut | GAATTCGCCACCATGGCAGCCGAGACTCGGAACGTGGCCGGAGCAGAGGCCCCACCGCCCCAGAAGCGCTACTACCGGCAACGTGCTCACTCCAACCCCATGGCGGACCACACGCTGCGCTACCCTGTGAAGCCAGAGGAGATGGACTGGTCTGAGCTATACCCAGAGTTCTTCGCTCCACTCACTCAAAATCAGAGCCACGATGACCCAAAGGATAAGAAAGAAAAGAGAGCTCAGGCCCAAGTGGAGTTTGCAGACATAGGCTGTGGCTATGGTGGCCTGTTAGTGGAACTGTCACCGCTGTTCCCAGACACACTTATTCTGGGTCTGGAGATCCGGGTGAAGGTCTCAGACTATGTACAAGACCGGATTCGGGCCCTACGCGCAGCTCCTGCAGGTGGCTTCCAGAACATCGCCTGTCTCCGTAGCAATGCCATGAAGCACCTTCCTAACTTCTTCTACAAGGGCCAGCTGACAAAGATGTTCTTCGCTTTCCCCGCGCCACATTTCAAGCGGACAAAGCACAAGTGGCGAATCATCAGTCCCACCCTGCTAGCAGAATATGCCTACGTGCTAAGAGTTGGGGGGCTGGTGTATACCATAACCGATGTGCTGGAGCTACACGACTGGATGTGCACTCATTTCGAAGAGCACCCACTGTTTGAGCGTGTGCCTCTGGAGGACCTGAGTGAAGACCCCGTTGTGGGACATCTAGGCACCTCAACTGAGGAGGGGAAGAAAGTTCTACGTAATGGAGGGAAGAATTTCCCAGCCATCTTCCGAAGAATACAAGATCCCGTCCTCCAGGCAGTGACCTCCCAAACCAGCCTGCCTGGTCACTGAGGATCC |
| **The sequences of Northern blot probes** | |
| **No.** | Sequences (5'-3') |
| **U6 snoRNA** | TGGAACGCTTCACGAATTTG |
| **proAGG** | CTCGTCCGGGATTTGAACCC |
| **ValAAC** | TGTTTCCGCCCGGTTTCGAA |
| **GlnCTG** | CAGAGTGCTAACCATTACACCATGGAACC |

**Table S6. Antibodies used in Western, Northwestern, and Northern Blot**

| **Antibodies** | **Source** | **Vendor** | **Identifier (Cat#)** | **Dilution** |
| --- | --- | --- | --- | --- |
| METTL1 | Rabbit | Proteintech  Abcam | 14994-1-AP  ab271063 | 1:4000 |
| WDR4 | Rabbit | Abcam | Ab169526 | 1:1000 |
| Cyclin B1 | Rabbit | CST | 12231S | 1:1000 |
| Cyclin D1 | Rabbit | CST | 55506S | 1:1000 |
| PCNA | Mouse | Proteintech | 60097-1-Ig | 1:5000 |
| YAP | Rabbit | CST | 14074S | 1:1000 |
| pYAP | Rabbit | CST | 13008S | 1:1000 |
| TAZ | Rabbit | CST | 59971S | 1:1000 |
| pTAZ | Rabbit | Abmart | TA4316 | 1:1000 |
| LATS1 | Rabbit | CST | 3477S | 1:1000 |
| pLATS1 | Rabbit | CST | 8654S | 1:1000 |
| LATS2 | Rabbit | Proteintech | 20276-1-AP | 1:1000 |
| pLATS2 | Rabbit | Affinity Biosciences | AF7440 | 1:1000 |
| Lamin B1 | Rabbit | CST | 13435S | 1:1000 |
| GAPDH | Rabbit | CST | 2118S | 1:1000 |
| β-Actin | Rabbit | CST | 4970L | 1:1000 |
| Anti-rabbit IgG HRP-linked Antibody | Rabbit | CST | 7074S | 1:10000 |
| Anti-mouse IgG HRP-linked Antibody | Mouse | CST | 7076S | 1:10000 |
| Mouse monoclonal 7-methylguanosine (m^7^G) | Mouse | MBL International | RN017M | 1:5000 |
| Mouse monoclonal anti-puromycin | Mouse | Millipore | MABE343 | 1:1000 |

**Table S7. Antibodies used in Immunohistochemistry staining and Immunofluorescence Staining**

| **Antibodies** | **Source** | **Vendor** | **Identifier (Cat#)** | **Dilution** |
| --- | --- | --- | --- | --- |
| METTL1 | Rabbit | Proteintech  Abcam | 14994-1-AP  ab271063 | 1:4000 |
| PCNA | Mouse | Proteintech | 60097-1-Ig | 1:4000 |
| Ki67 | Rabbit | Abcam  Novus Biological | ab15580  NB500-170 | 1:4000 |
| Brdu | Rabbit | CST | 5292S | 1:200 |
| YAP | Rabbit | Proteintech | 13584-1-AP | 1:200 |

**Table S8. Antibodies used in multiplex immunohistochemical staining**

| **Antibodies** | **Source** | **Vendor** | **Identifier (Cat#)** | **Dilution** |
| --- | --- | --- | --- | --- |
| METTL1 | Rabbit | Proteintech | 14994-1-AP | 1:4000 |
| PCNA | Mouse | Proteintech | 60097-1-Ig | 1:8000 |
| HNF4a | Rabbit | Abcam | Ab201460 | 1:10000 |
| CK19 | Rabbit | Abcam  Proteintech | ab52625  10712-1-AP | 1:4000 |
| α-SMA | Rabbit | Abcam | ab5694 | 1:4000 |

**Table S9. Primer sequences used in the study**

| **Target** | **Type** | **Primer sense** | **Sequence (5’-3’)** |
| --- | --- | --- | --- |
| *YAP-mouse* | qRT-PCR | F | TGAGATCCCTGATGATGTACCAC |
|  |  | R | TGTTGTTGTCTGATCGTTGTGAT |
| *YAP-human* | *qRT-PCR* | F | TAGCCCTGCGTAGCCAGTTA |
|  |  | R | TCATGCTTAGTCCACTGTCTGT |
| *TAZ-mouse* | *qRT-PCR* | F | ATGCCCCTCCATGTGAAGTG |
|  |  | R | GTGCCAACTAGGCCCATGAC |
| *TAZ-human* | *qRT-PCR* | F | CACCGTGTCCAATCACCAGTC |
|  |  | R | TCCAACGCATCAACTTCAGGT |
| *β-Actin-mouse* | *qRT-PCR* | F | CGCAGCCACTGTCGAGTC |
|  |  | R | GTCATCCATGGCGAACTGGT |
| *β-Actin-human* | *qRT-PCR* | F | CATGTACGTTGCTATCCAGGC |
|  |  | R | CTCCTTAATGTCACGCACGAT |
| *Areg-mouse* | *qRT-PCR* | F | AACTCTCCACAGGGGACTACG |
|  |  | R | CTTCTGTCTTGTTTTTCTTGGGC |
| *Birc5-mouse* | *qRT-PCR* | F | GAGGCTGGCTTCATCCACTG |
|  |  | R | ATGCTCCTCTATCGGGTTGTC |
| *Ctgf-mouse* | *qRT-PCR* | F | GACCCAACTATGATGCGAGCC |
|  |  | R | CCCATCCCACAGGTCTTAGAAC |
| *Ccnb1-mouse* | *qRT-PCR* | F | AGAGCTATCCTCATTGACTGGC |
|  |  | R | AACATGGCCGTTACACCGAC |
| *Cyr61-mouse* | *qRT-PCR* | F | TAAGGTCTGCGCTAAACAACTC |
|  |  | R | CAGATCCCTTTCAGAGCGGT |
| *Foxm1-mouse* | *qRT-PCR* | F | CAGAATGCCCCGAGTGAAACA |
|  |  | R | GTGGGGTGGTTGATAATCTTGAT |
| *METTL1-human* | *qRT-PCR* | F | GGCAACGTGCTCACTCCAA |
|  |  | R | CACAGCCTATGTCTGCAAACT |
| *METTL1-mouse* | *qRT-PCR* | F | GGCAACGTGCTCACTCCAA |
|  |  | R | CACAGCCTATGTCTGCAAACT |

**Table S10. Chemicals, peptides, and recombinant proteins**

|  | **Vendor** | **Identifier (Cat#)** |
| --- | --- | --- |
| RPMI Medium 1640 basic (1X) | GIBCO | C11875500BT |
| Fetal bovine serum | GIBCO | A3160801 |
| Penicillin-Streptomycin | GIBCO | 15140163 |
| Lipofectamine 3000 | Invitrogen | L3000015 |
| Polybrene | Solarbio | H8761 |
| Puromycin | Solarbio | P8230 |
| Trizol reagent | Life technologies | 15596018 |
| Cycloheximide | MedchemExpress | HY-12320 |
| MOPS | BioFroxx | 1173GR100 |
| MgCl2 | Aladdin | M113687 |
| NaCl | Aladdin | C111549 |
| KCl | Aladdin | P112134 |
| Triton™ X-100 | Sigma | 78787 |
| RNasin Ribonuclease Inhibitors | Invitrogen | 10777-019 |
| Heparin | Aladdin | D130947 |
| PMSF | Roche | 10837091001 |
| Benzamidine | Sigma | 12072 |
| Sodium borohydride (NaBH4) | Sigma | 452882 |
| Aniline | Sigma | 242284 |
| HEPES-KOH | Bestbio | BB-19324 |
| Dithiothreitol | Aladdin | D104859 |
| DAPI | Panovue | 0012100500 |
| TG520N | TG Medical | TGFT52100 |
| TG570N | TG Medical | TGFT57100 |
| TG620N | TG Medical | TGFT62100 |
| TG700N | TG Medical | TGFT70100 |
| HRP-Link secondary, Rabbit-Mouse | Panovue | 10013001050 |
| TSA | TG Medical | FFBN45 |
| REAL EnVision-HRP, Rabbit-Mouse | Agilent Technology | K5007 |
| Acetaminophen BioXtra, ≥99.0% | Sigma Aldrich | A7085 |
| Carbon Tetrachloride | GHTECH | 1.05033.026 |
| FITC | Biosharp | BL645 |
| Sirius Red Staining Kit | Phygene | PH1099 |
| Dual-Luciferase® Reporter Assay System | Promega | E1980 |
