# Supplementary figures and images for: METTL1‐Mediated M7G tRNA Modification Promotes Residual Liver Regeneration After Hepatectomy via Translational Control
Source: Adv Sci (Weinh). 2025 Dec 8;13(12):e07329. doi: 10.1002/advs.202507329 (PMC12948282; doi:10.1002/advs.202507329)

**Figure S1**

**A**

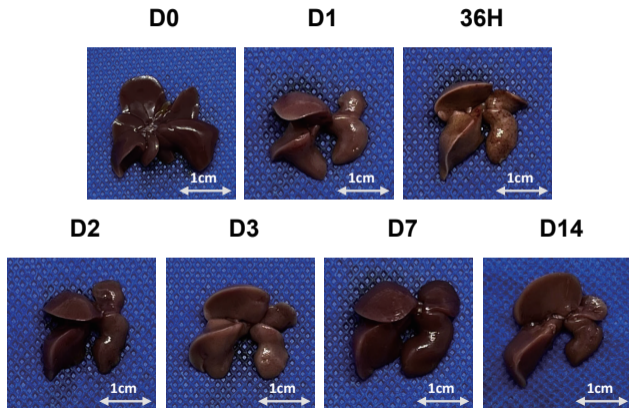

**B**

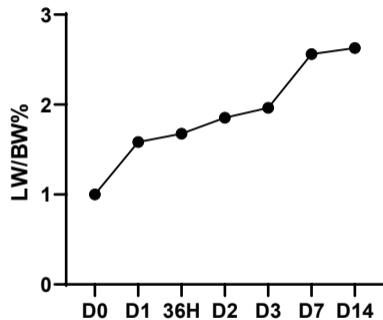

Supplement: Supplementary file 3 — Supporting Information [file ADVS-13-e07329-s002.pdf]

**Figure S2**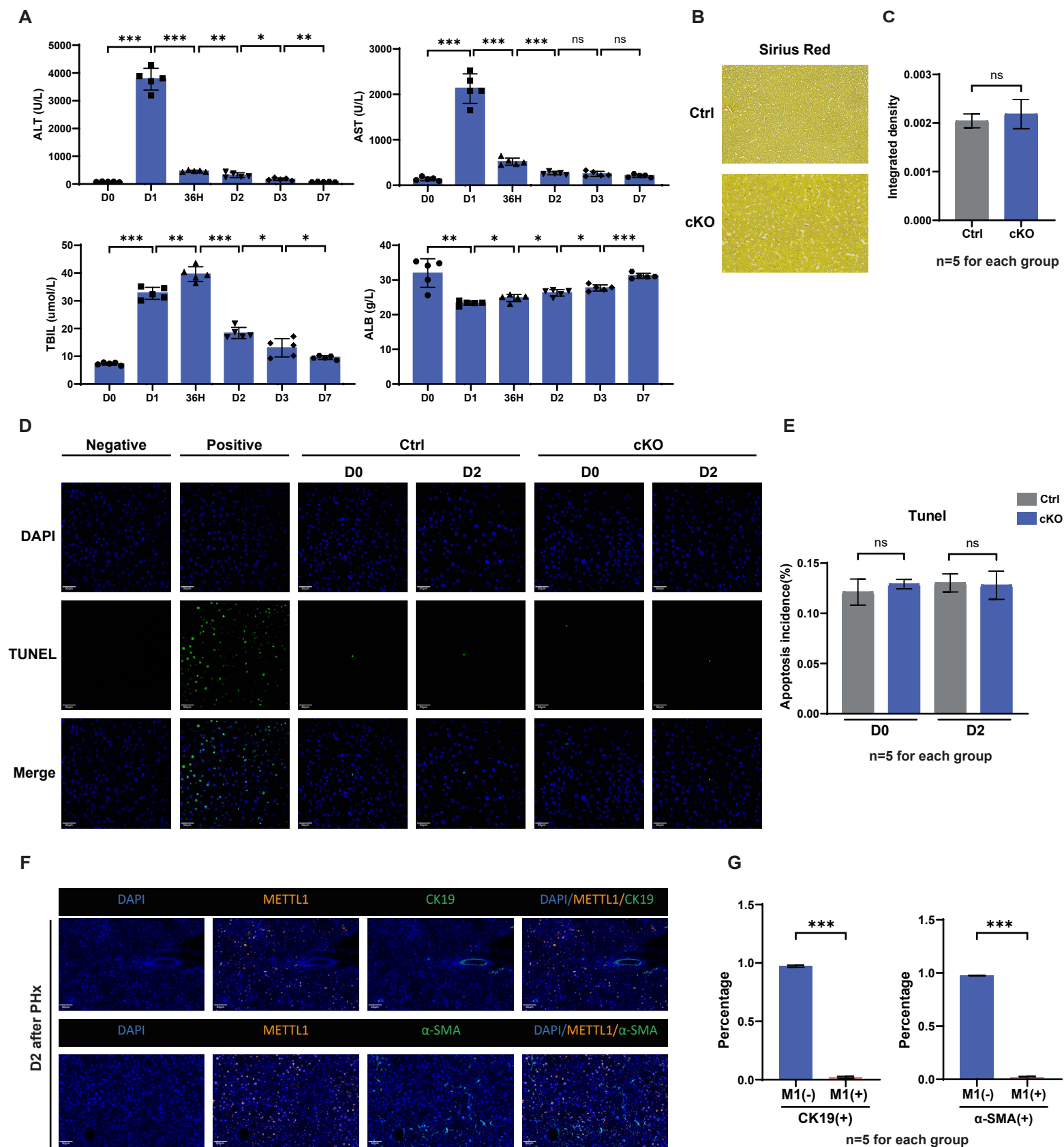

Supplement: Supplementary file 4 — Supporting Information [file ADVS-13-e07329-s003.pdf]

**Figure S3**

**A**

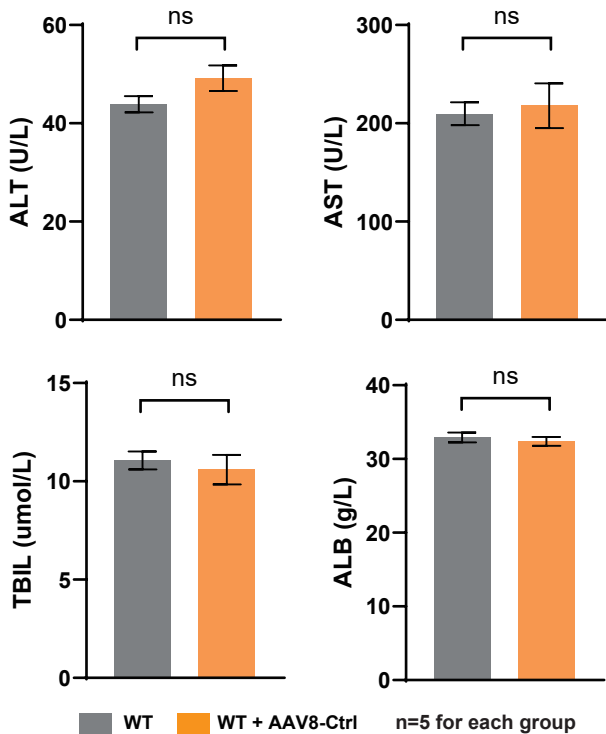

Supplement: Supplementary file 5 — Supporting Information [file ADVS-13-e07329-s008.pdf]

**Figure S4**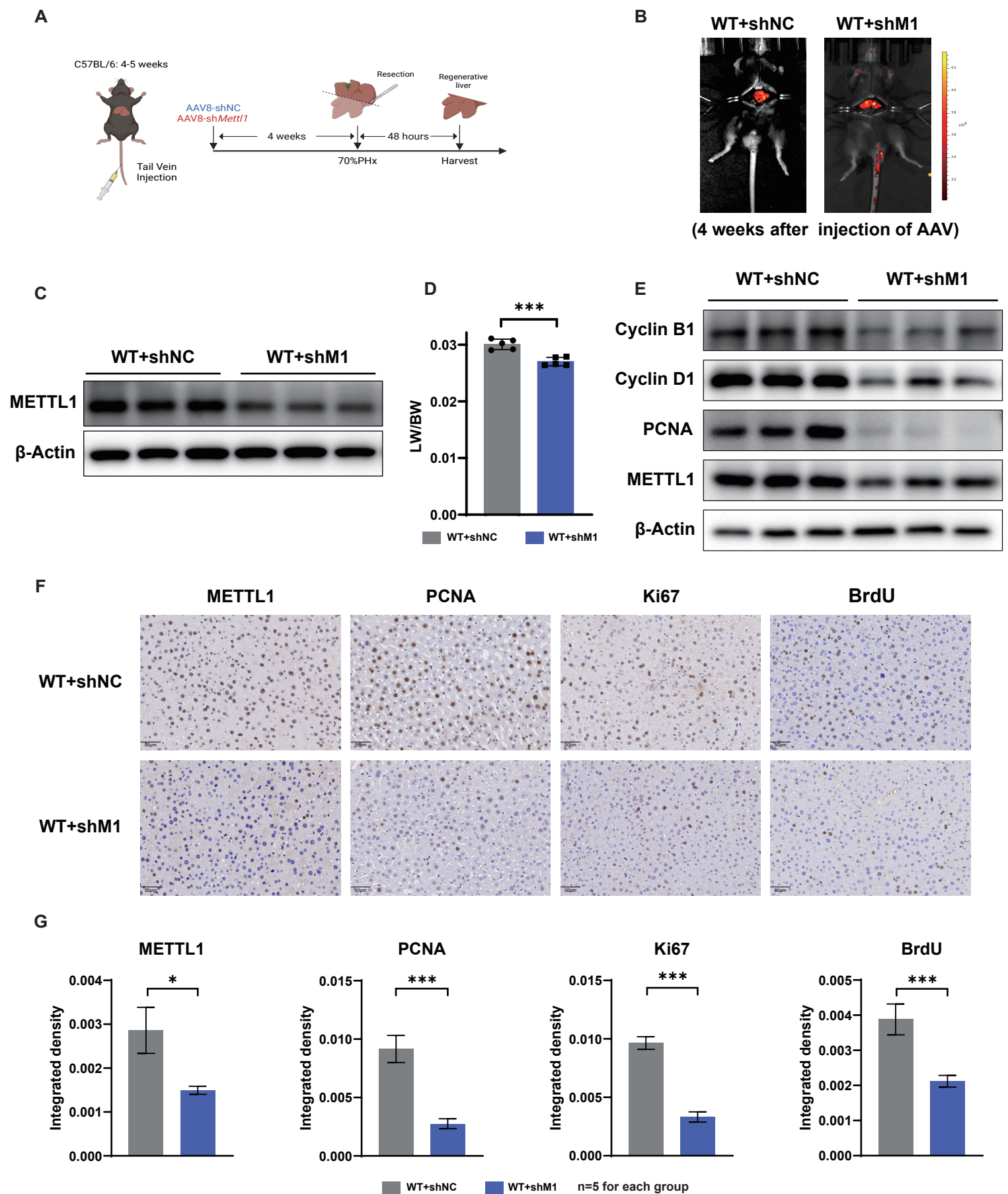

Supplement: Supplementary file 6 — Supporting Information [file ADVS-13-e07329-s006.pdf]

**Figure S5**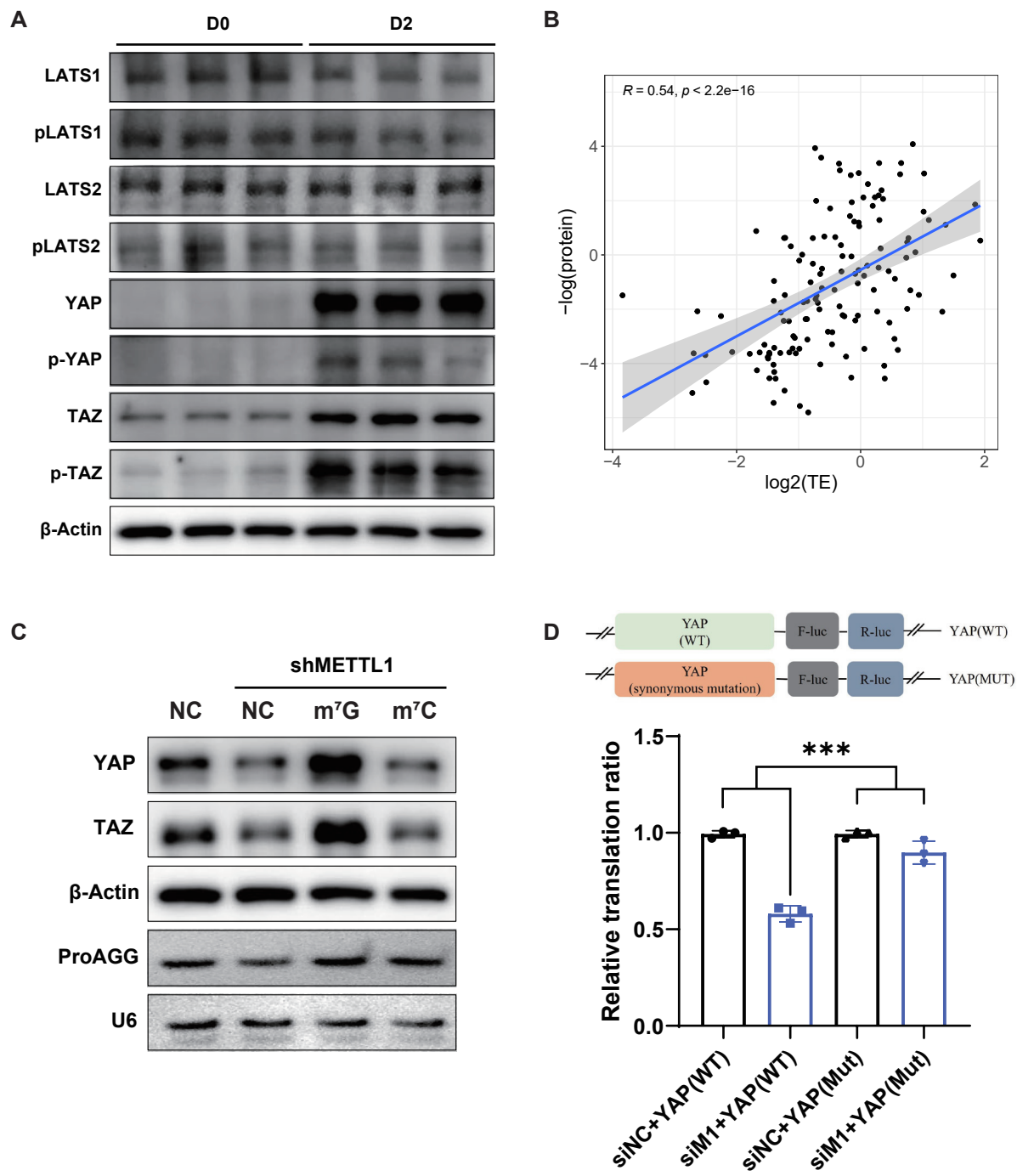

Supplement: Supplementary file 7 — Supporting Information [file ADVS-13-e07329-s001.pdf]

**Figure S6**

**A**

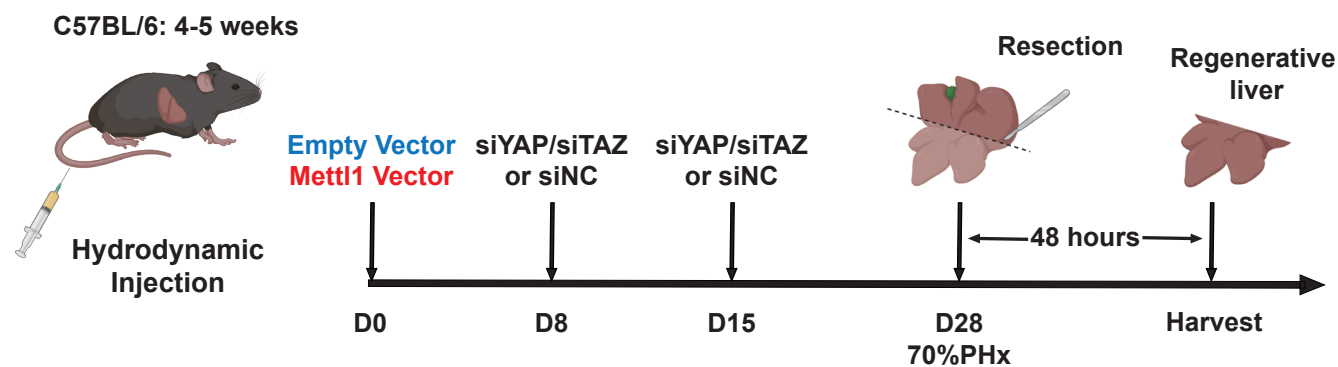

**B**

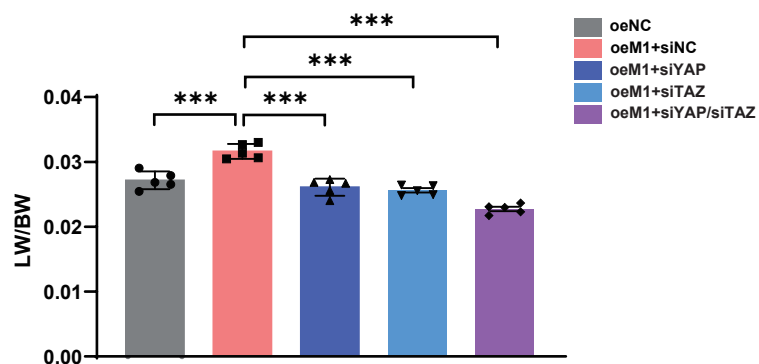

**C**

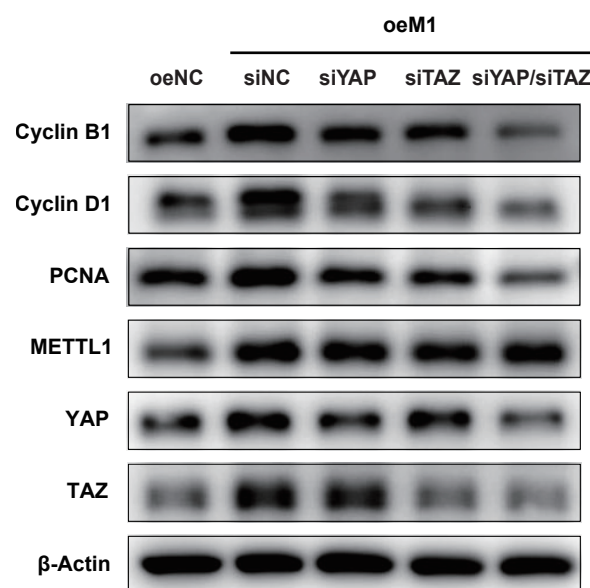

**D**

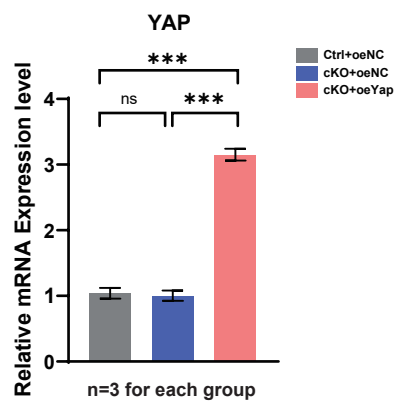

**E**

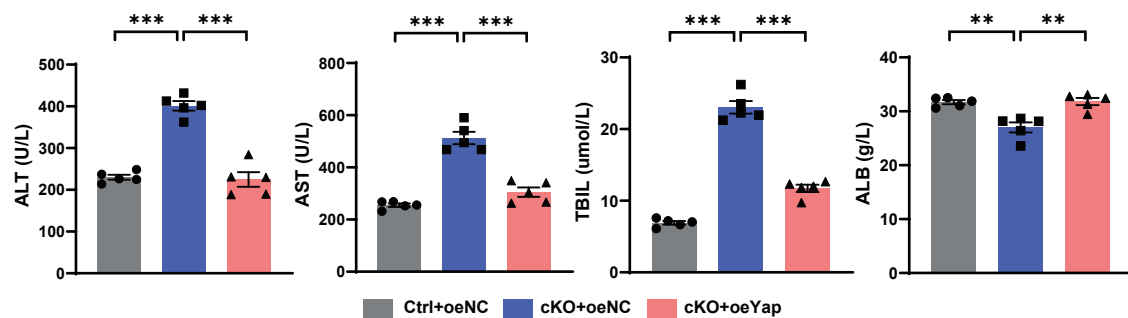

**F**

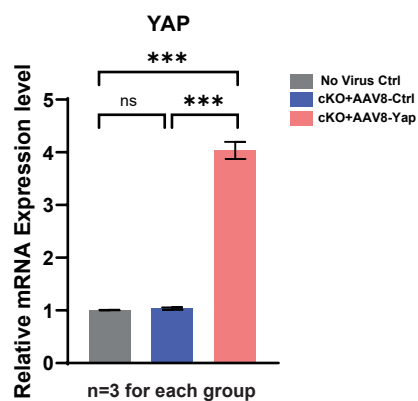

**G**

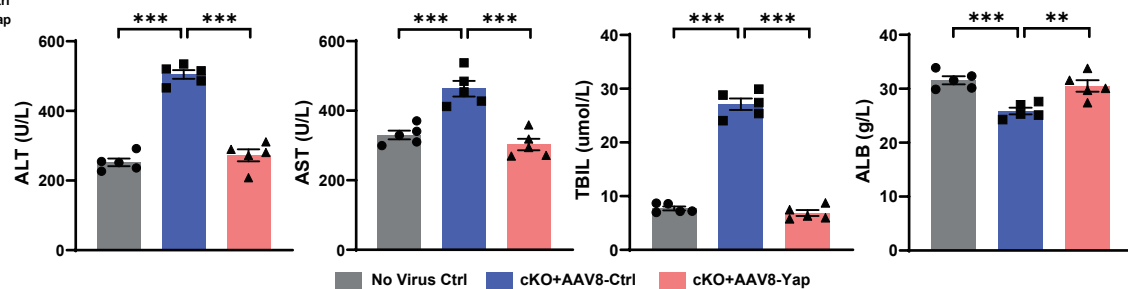

Supplement: Supplementary file 8 — Supporting Information [file ADVS-13-e07329-s009.pdf]

Figure S7

A

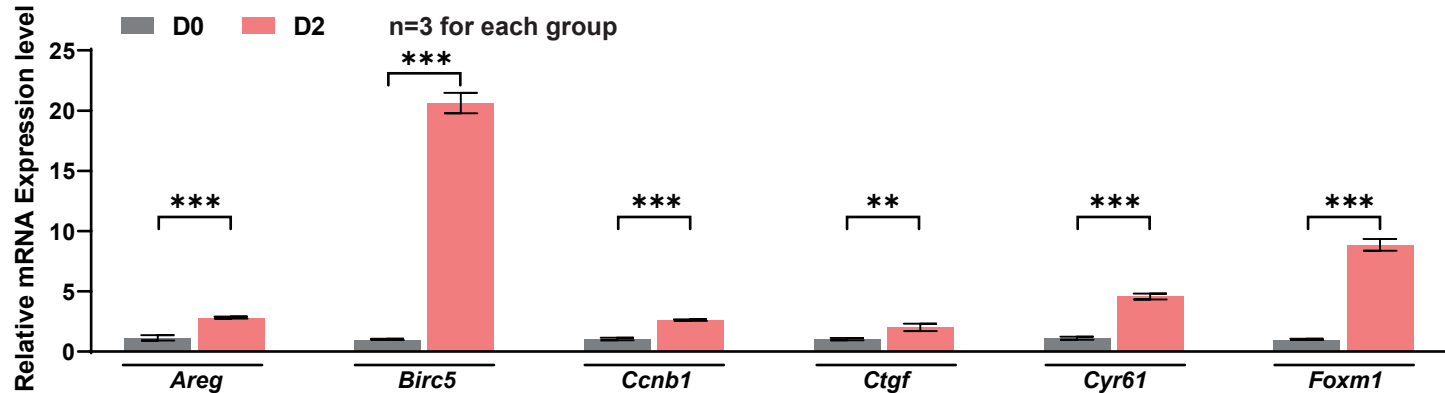

B

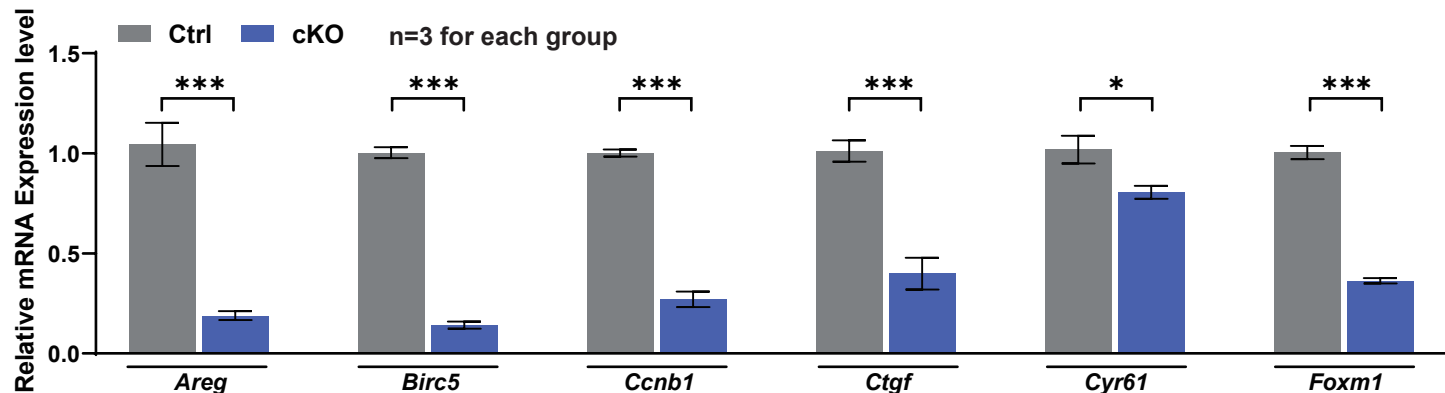

C

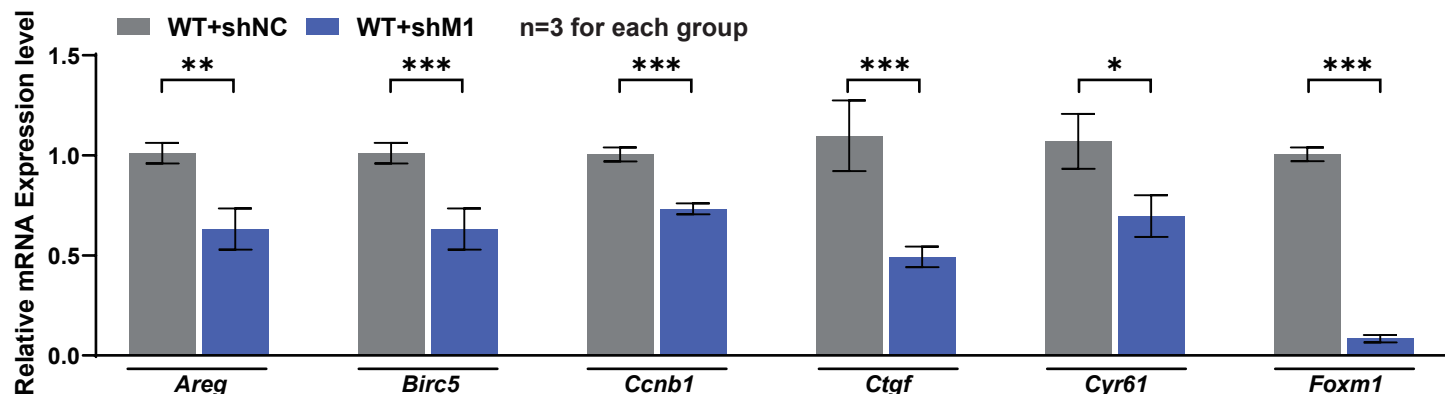

D

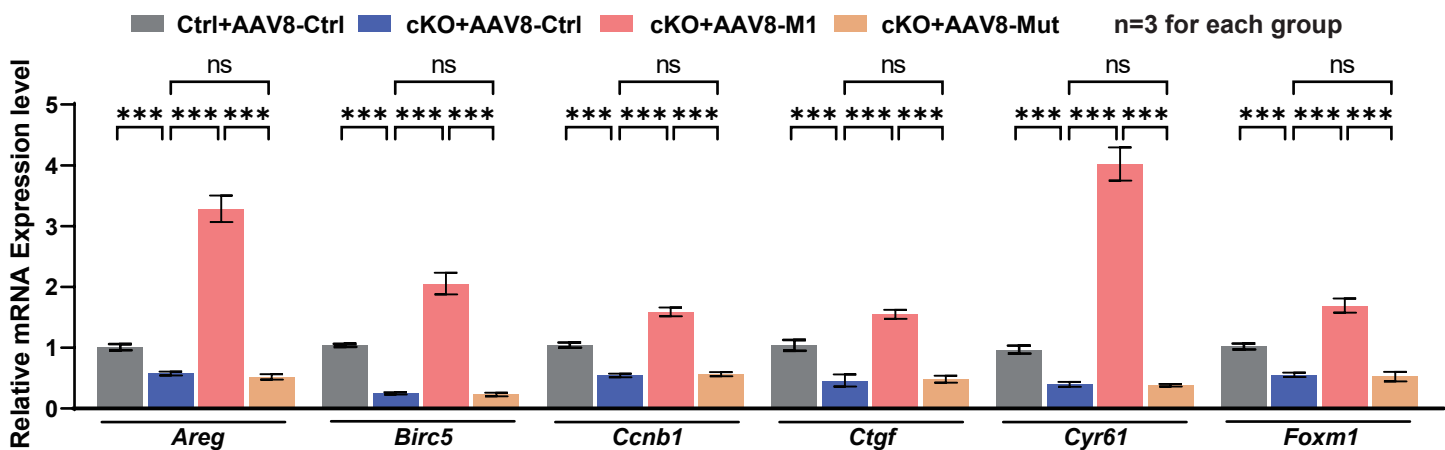

Supplement: Supplementary file 9 — Supporting Information [file ADVS-13-e07329-s005.pdf]

Figure S8

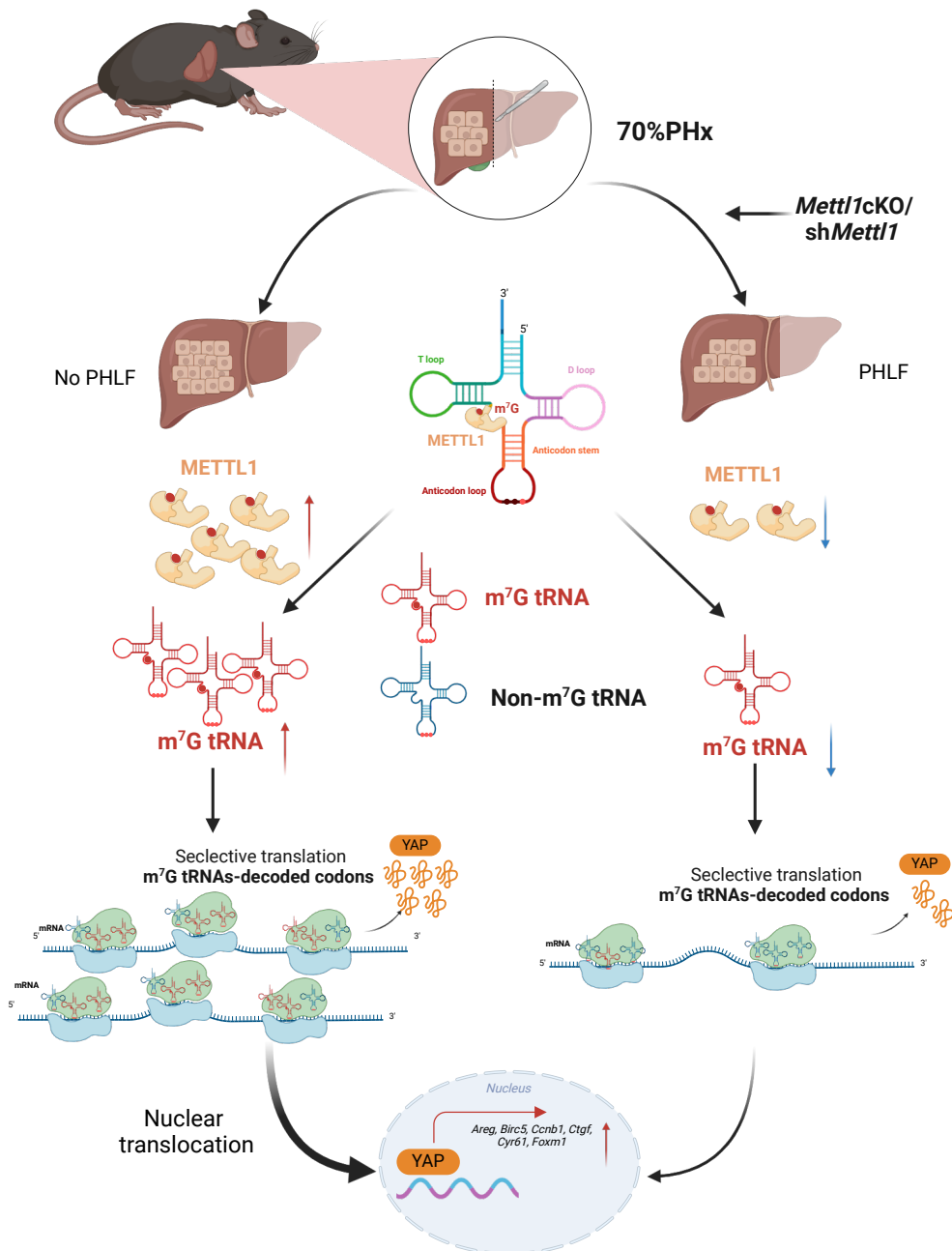

Supplement: Supplementary file 10 — Supporting Information [file ADVS-13-e07329-s010.pdf]

**Figure S9**

**A**

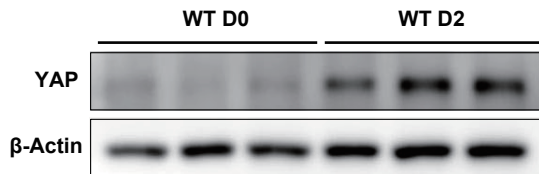

**B**

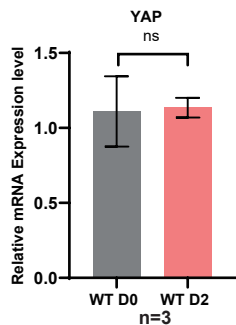

**C**

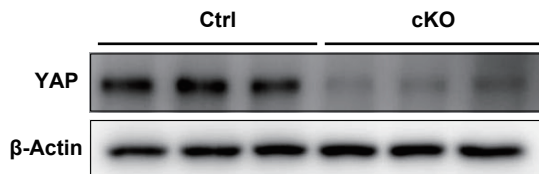

**D**

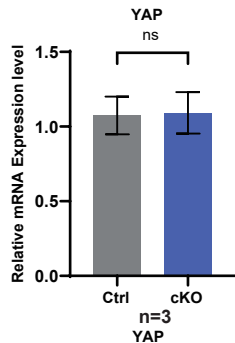

**E**

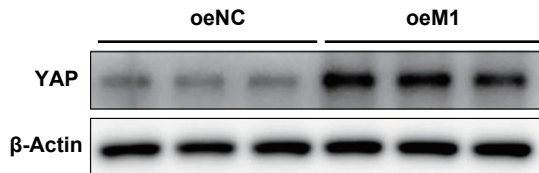

**F**

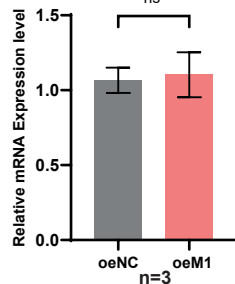

Supplement: Supplementary file 11 — Supporting Information [file ADVS-13-e07329-s007.pdf]
